# The impact of a tailored nutrition intervention delivered for the duration of hospitalisation on daily energy delivery for patients with critical illness (INTENT): a phase II randomised controlled trial

**DOI:** 10.1186/s13054-024-05189-3

**Published:** 2025-01-06

**Authors:** Emma J. Ridley, Michael Bailey, Marianne J. Chapman, Lee-anne S. Chapple, Adam M. Deane, Marlene Gojanovic, Alisa M. Higgins, Carol L. Hodgson, Victoria L. King, Andrea P. Marshall, Eliza G. Miller, Shay P. McGuinness, Rachael L. Parke, Eldho Paul, Andrew A. Udy, The Australian, New Zealand Intensive Care Society Clinical Trials Group, Emma J. Ridley, Michael Bailey, Marianne J. Chapman, Lee-anne S. Chapple, Adam M. Deane, Marlene Gojanovic, Alisa M. Higgins, Carol L. Hodgson, Victoria L. King, Andrea P. Marshall, Eliza G. Miller, Shay P. McGuinness, Rachael L. Parke, Eldho Paul, Andrew A. Udy, Farisha Ali, Rebecca Baskett, Magdalena Butler, Keri-Anne Cowdrey, Eileen Gilder, Lydia Gillan, Su-Zahn Koorts, Brittany Mason, Ellie McMahon, Shay McGuinness, Karina O’Connor, Rachael Parke, Melissa Robertson, Samantha Ryan, Anna Small, Andrew Xia, Megan Berner, Christine Choong, Glenn Eastwood, Kate Hamilton, Daryl Jones, Leah Peck, Helen Young, Lauren Ballantyne, Catherine Boschert, Cameron Knott, Bridget Roberts, Julie Smith, Zoe Flick, Kalpesh Gandhi, Barbara Hannah, Yvonne Li, Kiran Nand, Treena Sara, Sylvia Wei, Lina Briek, Graeme Duke, Kym Gellie, Stephanie Hunter, Nicole Robertson, Jonathan Barrett, Brydie Cleeve, Caroline Guille, Gabrielle Hanlon, Sarah Jelly-Butterworth, Julie O’Donnell, Carmel Zoanetti, Zhoe Coram, Karen Edis, Mike Gomez, Alice Goodman, Kavi Haji, Jenny Moss, Jodie Prendergast, Janet Tam, Ravindranath Tiruvoipati, Fiona Turnbull, Julie Cussen, Maimoonbe Gough, Sarah Lovelock, Lisa Mahoney, Andrea Marshall, David Pearson, Mandy Tallott, Emily Ainslie, Kate Flynn, Kerri Gordon, Tim Stewart, Larissa Telfer, Victoria Williams, Vishwanath Biradar, Hanaa Chahine, Edda Jessen, Matia Kapsambelis, Melissa Lydyard, Ashlee Martin, Julie Puccini, Natalie Soar, Leah Sommerfield, Bronwyn Bartholomew, Peter Collins, Claire Filet, Mackenzie Finnis, Chloe Jobber, Katherine Jongebloed, Isabel Anne Leditschke, Kimberley Browning, Jennifer Chang, Dinu Girijadevi, Areege Hussein, Vivian Lai, Rima Song, Tony Williams, Oshara de Silva, Ashlee Gervasoni, Carolyn Hall, Lauren Hanna, Sheree Phillips, Yahya Shehabi, Rebecca Gresham, Matin Jamei, Sheeja Joy, Julie Lowrey, Kristy Masters, Ian Seppelt, Wendy Tu, Christina Whitehead, Tina Aboltins, Hayley Collins, Rachael Evans, Angaj Ghosh, Simone Said, Vivian Tsang, Lynette De Groot, Ra’eesa Doola, Meg Harward, Cassie Jones, Josephine Mackay, Jason Meyer, Tahnie Takefala, James Walsham, Stuart Baker, Anthony Khoo, Shannon Lewis, Alyce Nissen, Alexis Tabah, Alicia Wiese, Lewis Campbell, Miriam Chin, Rebecca Garcia, Kirsty Smyth, Annabel Thallon, Emma Bidgood, Jessica Browne, Kathleen Byrne, Adam Deane, Kate Fetterplace, Hilda Griffin, Sarah Phillips, Kym Wittholz, Jasmin Board, Peta Bretag, Aidan Burrell, Adam Cunningham, Dashiell Gantner, Ramez Hanna, Kate Lambell, Karina Lay, Elisa Licari, Lee Lin Loh, Emma Martin, Phoebe McCracken, Jenna Obeid, Caitlin Rabel, Peter Thanhauser, Andrew Udy, Chloe Vadiveloo, Cyndi Wong, Meredith Young, Cameron French, Greta Hollis, Adrian Powlesland, Kiran Shekar, Marion Vasudevan, Emma Whitmore, Tennealle Direen, Martine Hatzi, Cathy Kurenda, Sandra Peake, Amber Thatcher, Patricia Williams, Michelle Horton, Nima Kakho, Matthew Maiden, Tania Salerno, Jemma Trickey

**Affiliations:** 1 Australian and New Zealand Intensive Care Research Centre, School of Public Health and Preventive Medicine, Department of Epidemiology and Preventative Medicine, Monash University, 553 St Kilda Road, Melbourne, VIC Australia; 2 Nutrition Department, The Alfred Hospital, Melbourne, VIC Australia; 3 Adelaide Medical School, University of Adelaide, Adelaide, South Australia Australia; 4 Intensive Care Unit, Royal Adelaide Hospital, Adelaide, South Australia Australia; 5 Department of Critical Care, Melbourne Medical School, The University of Melbourne, Melbourne, Australia; 6 Intensive Care Unit, Alfred Hospital, Melbourne, VIC Australia; 7 Department of Critical Care, The George Institute for Global Health, Sydney, NSW Australia; 8 Department of Critical Care, The University of Melbourne, Melbourne, VIC Australia; 9 Gold Coast Hospital and Health Service, Southport, QLD Australia; 10 School of Nursing and Midwifery, Griffith University, Gold Coast Campus, Southport, QLD Australia; 11 Cardiothoracic and Vascular Intensive Care Unit, Auckland City Hospital, Auckland, New Zealand; 12 School of Nursing, The University of Auckland, Auckland, New Zealand; 13 Auckland City Hospital Cardiothoracic and Vascular Intensive Care Unit, Auckland, New Zealand; 14 Austin Hospital, Melbourne, VIC Australia; 15 Bendigo Health, Bendigo, VIC Australia; 16 Blacktown Hospital, Blacktown, NSW Australia; 17 Box Hill Hospital, Melbourne, VIC Australia; 18 Epworth Hospital Richmond, Melbourne, VIC Australia; 19 Frankston Hospital, Frankston, VIC Australia; 20 Gold Coast University Hospital, Southport, QLD Australia; 21 Ballarat, VIC Australia; 22 Lyell McEwin Hospital, Adelaide, SA Australia; 23 Mater Misericordiae Ltd, Brisbane, QLD Australia; 24 Middlemore Hospital, Auckland, New Zealand; 25 Monash Medical Centre, Melbourne, VIC Australia; 26 Nepean Hospital, Sydney, NSW Australia; 27 Northern Hospital, Melbourne, VIC Australia; 28 Princess Alexandra Hospital, Brisbane, QLD Australia; 29 Redcliffe Hospital, Redcliffe, QLD Australia; 30 Royal Darwin Hospital, Darwin, NT Australia; 31 Royal Melbourne Hospital, Melbourne, VIC Australia; 32 The Alfred, Melbourne, VIC Australia; 33 The Prince Charles Hospital, Brisbane, QLD Australia; 34 The Queen Elizabeth Hospital, Adelaide, SA Australia; 35 University Hospital Geelong, Geelong, VIC Australia

## Abstract

**Background:**

Nutrition interventions commenced in ICU and continued through to hospital discharge have not been definitively tested in critical care to date. To commence a program of research, we aimed to determine if a tailored nutrition intervention delivered for the duration of hospitalisation delivers more energy than usual care to patients initially admitted to the Intensive Care Unit (ICU).

**Methods:**

A multicentre, unblinded, parallel-group, phase II trial was conducted in twenty-two hospitals in Australia and New Zealand. Adult patients, requiring invasive mechanical ventilation (MV) for 72–120 h within ICU, and receiving < 80% estimated energy requirements from enteral nutrition (EN) were included. The intervention (tailored nutrition) commenced in ICU and included EN and supplemental parenteral nutrition (PN), and EN, PN, and/or oral nutrition after liberation from MV, and was continued until hospital discharge or study day 28. The primary outcome was daily energy delivery from nutrition (kcal). Secondary outcomes included duration of hospital stay, ventilator free days at day 28 and total blood stream infection rate.

**Main results:**

The modified intention to treat analysis included 237 patients (n = 119 intervention and n = 118 usual care). Baseline characteristics were balanced; the median [interquartile range] intervention period was 19 [14–35] and 19 [13–32] days in the tailored nutrition and usual care groups respectively. Energy delivery was 1796 ± 31 kcal/day (tailored nutrition) versus 1482 ± 32 kcal/day (usual care)—adjusted mean difference 271 kcal/day, 95% CI 189–354 kcal. No differences were observed in any secondary outcomes.

**Conclusions:**

A tailored nutrition intervention commenced in the ICU and continued until hospital discharge achieved a significant increase in energy delivery over the duration of hospitalisation for patients initially admitted to the ICU.

**Trial registration** ClinicalTrials.gov Identifier NCT03292237. First registered 25th September 2017. Last updated 10th Feb 2023.

**Supplementary Information:**

The online version contains supplementary material available at 10.1186/s13054-024-05189-3.

## Introduction

Severe critical illness induces significant catabolism, causing muscle wasting and weight loss [1]. Although it is hypothesised that augmented nutrition may prevent muscle wasting and weight loss, definitive benefits remain unclear [1, 2]. Successful interventions to augment energy delivery in critical illness include supplemental parenteral nutrition (PN) and higher energy enteral nutrition (EN); however, no benefit has been observed, and one study showed harm [3, 4]. This lack of benefit aligns with many other trials investigating various critical care nutrition interventions [5–12]. With the exception of the EPaNIC trial, all intervention durations were short (5–7 days), in the early acute phase of illness, and in heterogeneous populations [5–7, 9–12]. Metabolic alterations in early critical illness, including insulin and anabolic resistance, may limit the effective utilization of nutrition, possibly explaining the lack of benefit [1, 2].

The European Society for Clinical Nutrition and Metabolism categorizes critical illness into “acute early”, “acute late”, and “recovery” stages, recommending tailored and progressive nutrition based on the phase and acknowledges changing nutritional requirements throughout illness [8]. The latest nutrition guideline from the American Society of Parenteral and Enteral Nutrition identifies the need to describe nutrition intake for the entire period of outcome observation in critical illness [13]. Nutrition interventions continued for the duration of hospitalisation have shown clinical benefits for non-critically ill patients [14]; however, practical challenges such as gastrointestinal intolerance and fasting for procedures often limit energy delivery to less than 50% of recommendations during the early phase of critical illness and may prevent prolonged nutrition enhancement in critically ill populations [15]. Moreover, observational data from various geographical regions indicate suboptimal nutrition provision in the late and post-intensive care unit (ICU) period, with no established strategies for extension of nutrition interventions from ICU to hospital discharge [16–22].

To begin to address this gap, the aim of the Intensive Nutrition Therapy comparEd to usual care iN criTically ill adults (INTENT) trial was to establish if a tailored nutrition intervention provided throughout hospitalisation could deliver more energy compared to usual care in patients initially admitted to the ICU.

## Methods

This study is reported according to the CONSORT statement, with a priori registration (NCT03292237) and publication of the protocol and statistical analysis plan [23].

### Trial design

This was a multicentre, prospective, unblinded, parallel, phase II randomised controlled trial (RCT) with patients allocated 1:1 to a tailored nutrition intervention or usual care.

### Participants

Patients aged ≥ 18 years and between 72 and 120 h of their index ICU admission were screened for eligibility. Eligible patients required invasive mechanical ventilation (MV), had one or more organ system failure, a central line for PN provision (if so allocated to tailored nutrition) and had received < 80% estimated energy requirements from EN in the previous 24 h (Supplemental Digital Content Additional File 1). The study was conducted in 23 ICUs within Australia and New Zealand (ANZ) and was endorsed by the Australian and New Zealand Intensive Care Society Clinical Trials Group (Additional File 2). Ethics approval was obtained from the Alfred Hospital Ethics Committee (HREC/18/Alfred/101) and the Human Research Ethics Committee of the Northern Territory Department of Health (2019–3372) in Australia and the New Zealand Central Health and Disability Ethics Committee (18/NTA/222/AM01) in New Zealand. The original protocol was approved on 31st July 2018, with a subsequent minor protocol amendment of editorial changes for clarity approved on 8th January 2020 (Additional File 3).

### Interventions

**Fig. 1:**
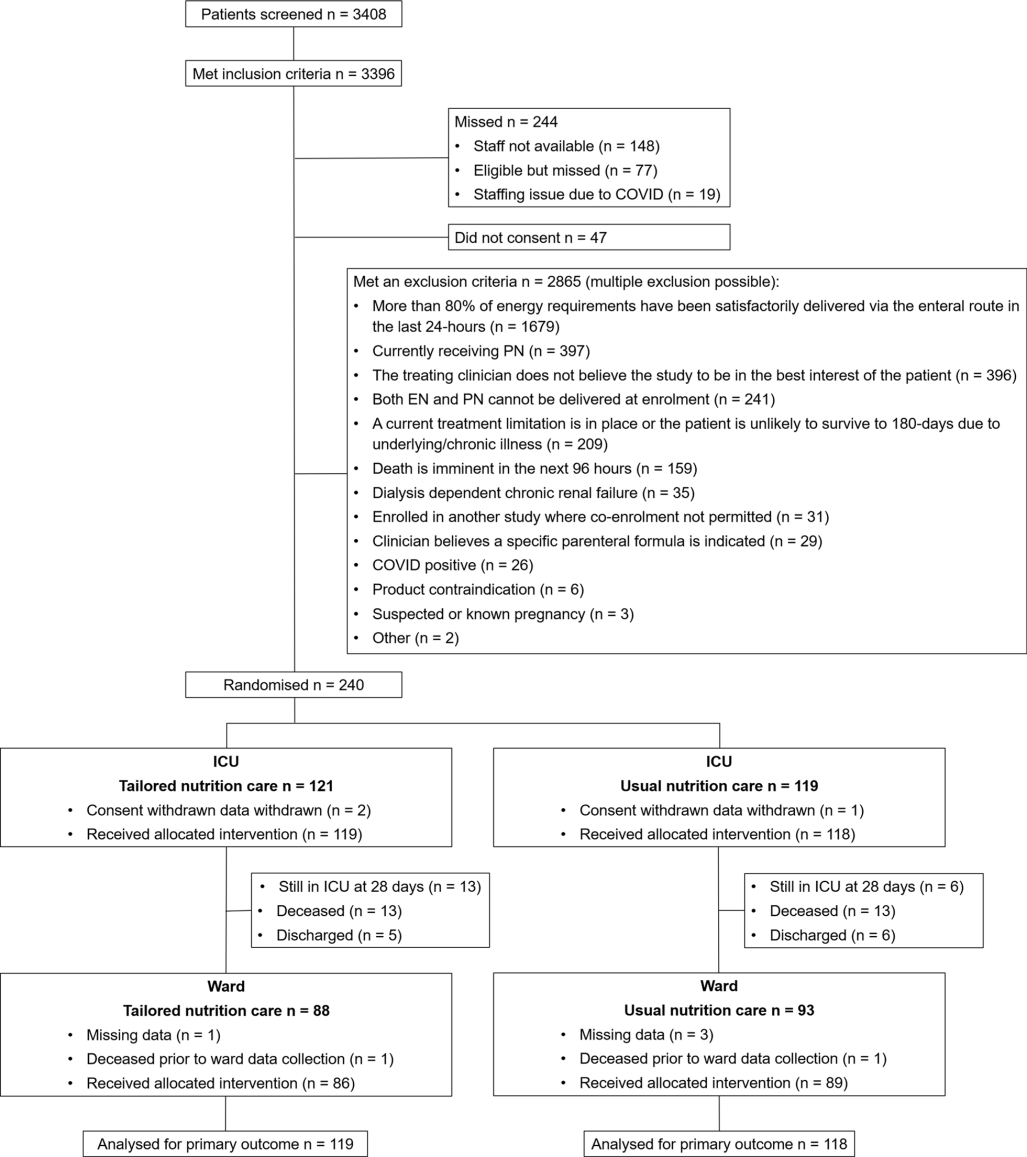
CONSORT diagram

The intervention group received tailored nutrition from randomisation in ICU to hospital discharge or study day 28, aiming for energy provision between 80 and 100% of predicted requirements at all times (whilst avoiding overfeeding, defined as ≥ 110% of the study energy requirement). In ICU, supplemental parenteral nutrition (PN) was provided whenever daily energy provision was < 80% of the study energy requirement. This was followed with tailored nutrition care, delivered by an INTENT study dietitian of oral, EN or PN according to clinical indication, until hospital discharge or day 28. Full study processes within ICU and the ward are outlined in detail in Fig. 1.

The interventional PN was Olimel N12E with a multi-trace element solution (10 ml), multi-vitamin (Cernevit, Baxter Healthcare Corporation, 5 ml) and ascorbate (125 mg) for stability, manufactured and supplied by Baxter Healthcare Corporation (composition, Additional File 4). Once oral intake commenced, two study oral nutrition supplements (Fortisip Compact Protein or Forticreme Complete) were prescribed at a recommended dose of 60 ml four times per day (composition, Additional File 5).

### Intervention process

#### Day of randomisation:

Interventional PN was commenced via a central venous catheter within 2 h of randomisation based on the amount of energy received from EN in the previous 24 h, to achieve 80–100% of the study energy requirement (Additional File Fig. 2a).

**Fig. 2:**
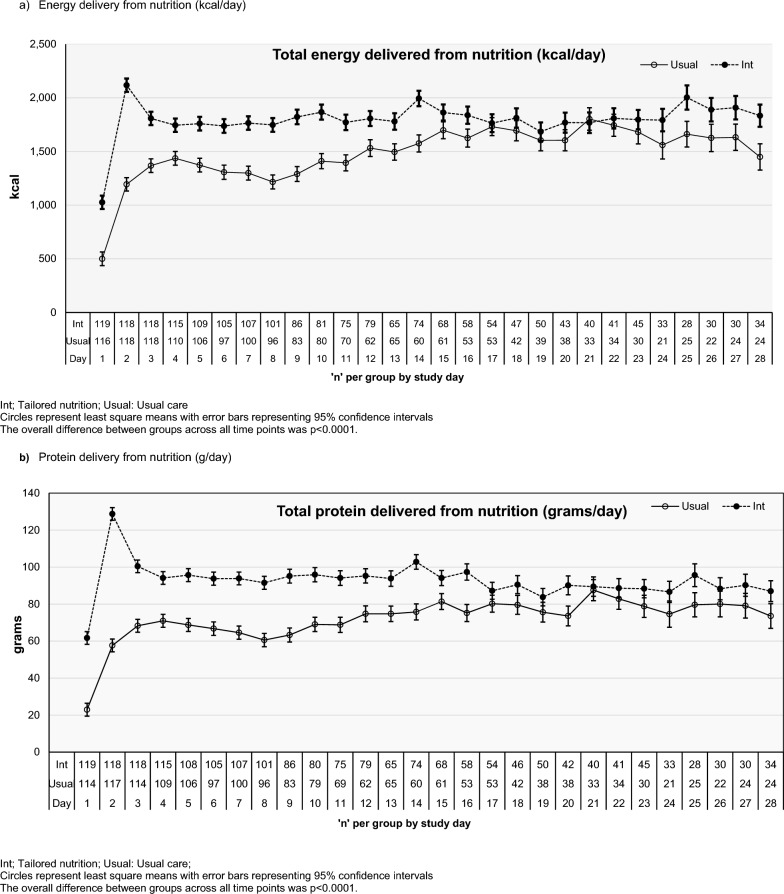
Delivery of nutrition over 28-day study period. **A** Energy delivery from nutrition (kcal/day), **b** Protein delivery from nutrition (g/day)

#### ICU

From study day 2 until ICU discharge (or removal of the central line according to the decision of the treating clinical team), intervention PN rates for the next 24 h were based on energy delivered from EN, PN, oral nutrition and non-nutrition energy sources (including glucose ≥ 25% and propofol) in the previous 24 h, with three rates possible: off, 10 kcal/kg calculated body weight (CBW)/day, or 20 kcal/kg CBW/day. If EN was interrupted for ≥ 2 h, PN was provided at the 20 kcal/kg CBW/day rate (Additional File Fig. 2b).

#### Ward

Participants were reviewed daily by a study dietitian to ensure the nutrition management plan was appropriate (with a minimum of three formal nutrition reviews for data collection per week, Additional File Fig. 1).

### Usual care process

Usual nutrition care was the comparator, with all aspects delivered according to local hospital protocols. In cases of absolute contraindications to EN or prolonged delivery issues for patients allocated to usual care, PN was allowed in the ICU (using the intervention PN to standardise across group) after attempts to improve EN had occurred. Indications for PN and processes to optimise EN were according to usual care processes at each individual site.

### Procedures common to both groups

Individual energy requirements were set at 25 kcal/kg CBW/day throughout the ICU stay (Additional File 6). CBW equalled actual body weight for participants < 65 years with a BMI < 25 kg/m^2^ (or if ≥ 65 years, < 30 kg/m2) or adjusted body weight for overweight or obese patients (Additional File 7). Upon transfer to the hospital ward, energy requirements could continue or be re-evaluated by clinical staff.

In ICU and on the ward, EN formula selection, protein requirement, blood glucose control, timing of nasogastric tube (NGT) removal/reinsertion and initiation of oral intake adhered to local hospital protocols. Strategies were suggested to avoid overfeeding if all nutrition sources delivered > 110% of the participants’ study energy requirement in ICU (Additional File 7). Upon starting oral diet, food record charts were requested for both groups. Continuation of PN on the ward was determined by local site indications for both groups, using the usual formula/s available at the site.

### Outcomes

The primary outcome was energy delivered from nutrition therapy in kcal/day up to hospital discharge or day 28. Secondary outcomes included protein intake (g/day) to hospital discharge or day 28, energy (kcal/day) and protein intake (g/day) by ICU and ward location, duration of hospital stay, ventilator free days to day 28 and total blood stream infection rate (hospital admission to day 28). Tertiary outcomes included duration of ICU stay, duration of invasive MV to day 28, ICU mobility scale at ICU discharge, number of blood stream infections to day 28, time to first blood stream infection, in-hospital and 28-day mortality, and weight at hospital discharge (kg). Central Line Associated Blood Stream Infection (CLABSI) was added as a tertiary outcome of interest post-hoc. Outcome variables are defined in Additional File 8 and adverse events and protocol deviations in Additional File 9.

### Randomisation

The randomisation schedule was generated by the study statistician, stratified by site in permuted blocks of variable size (2 and 4). Randomisation and allocation concealment occurred via a dedicated, secure, password protected internet-based website designed by Research Path Pty Ltd.

As a phase II trial, no interim analysis or feasibility stopping rules were pre-specified. A Data Safety Monitoring Committee (DSMC) advised as required (Additional File 2).

### Statistical methods:

Sample size was determined from our pilot RCT where the mean ± standard deviation (SD) energy delivered to the usual care arm throughout hospital stay was 1540 ± 410 kcal/day [25]. With 190 subjects in total, this study has 95% power (2-sided p-value of 0.05) to detect a clinically acceptable difference of 15% (215 kcal/day). To account for possible loss to follow-up, this figure was inflated by 20% to total 240 patients.

Analysis was performed on a modified intention to treat basis excluding only participants who withdrew consent. Potential baseline imbalance between groups was determined using Chi-square tests for equal proportion, Student’s t-test was used for normally distributed outcomes, and Wilcoxon rank-sum tests otherwise, with results reported as numbers (percentages), means ± SD or medians [interquartile range (IQR)], respectively.

Longitudinal analysis of daily total energy (and protein) intake was performed using hierarchical mixed linear modelling with patients nested within sites and both patients and sites treated as random effects, fitting main effect for location (ICU or ward), treatment and time, and an interaction between the latter to determine if treatment effects varied over time. Results were reported as least square means ± standard errors and mean differences (95% Confidence Interval (CI)). Heterogeneity across locations was further determined by fitting an interaction between treatment and location. Sensitivity to known covariates was performed using covariate adjustment for a priori determined variables (age, BMI, clinical frailty score, admission diagnosis, and illness severity (APACHE II)).

Times to extubation, ICU and hospital discharge were analysed using Fine and Gray frailty models to account for the competing risk of death, with results reported as sub-distributional hazard ratios (95% CI) and presented as cumulative incidence graphs. Patient survival was analysed using Cox-proportional hazards regression including clustering for site with results reported as Hazard ratios (95% CI) and presented as Kaplan Meier survival curves. Subgroup analysis was performed for the primary outcome on seven subgroups determined at baseline (Additional File 8) [23]. Analyses were performed using SAS software, version 9.4 (SAS Institute) and a two-sided p-value of 0.05 was used to indicate statistical significance. No adjustment was made for multiple comparisons with all non-primary outcomes considered as hypothesis generating. Prior to completion, a detailed analysis plan was published (further analysis details, Additional File 8 [23]).

## Results

From October 15, 2018, to January 31, 2023, 240 patients were randomised from 22 sites. Two patients in tailored nutrition and one in usual care withdrew consent for data, resulting in 237 patients for the intention-to-treat analysis (119 in tailored nutrition, 118 in usual care, Fig. 1 and Additional File Table 1). Baseline characteristics were comparable (Table 1 and Additional File Table 1), with the majority of patients admitted with a cardiovascular diagnosis (n = 93 (38%)). Cardiac surgery occurred in 24/119 (20%) patients in tailored nutrition and 25/118 (21%) in usual care. In keeping with the inclusion window of between 72 and 120 h of ICU admission, the median time from hospital admission to randomisation and trial period (hospital length of stay) was approximately 4 days and 19 days, respectively.

**Table 1:** Baseline participant characteristics

	Tailored nutrition (n = 119)	Usual care (n = 118)
Age, years	55 ± 17	57 ± 16
Sex, male, n (%)	83 (70%)	78 (66%)
Calculated body weight, kg	84 ± 15	82 ± 15
BMI, kg/m^2^	32 ± 8	30 ± 8
APACHE II score	16 ± 7	18 ± 6
*APACHE III diagnosis code, n (%)*		
Cardiovascular	44 (37%)	49 (42%)
Respiratory	20 (17%)	19 (16%)
Trauma	16 (13%)	17 (14%)
Sepsis	12 (10%)	17 (14%)
Neurological	13 (11%)	7 (6%)
Gastrointestinal	11 (9%)	4 (3%)
Metabolic	1 (1%)	3 (3%)
Musculoskeletal	2 (2%)	1 (1%)
Renal	0 (0%)	1 (1%)
RRT commenced prior to randomisation, n (%)	29 (24%)	34 (29%)
Baseline SOFA score	9 [6–11]	9 [6–11]
NUTRIC score	4 [3–5]	4 [3–5]
Clinical frailty Score	3 [2–4]	3 [2–4]
Study energy requirement, kcal/day	2089 ± 368	2034 ± 372
Clinician estimated protein requirement, g/day	104 ± 17	104 ± 21
Daily energy received from hospital admission to randomisation from all sources, kcal/day	801 ± 473	758 ± 493
Time from hospital admission to randomisation, days	4 [4–5]	4 [4–5]

Continuous normally distributed data are presented as mean ± standard deviation (SD), otherwise as median [interquartile range] (IQR). Baseline SOFA was assessed using the most deranged physiological values within 24 h of randomisation Study energy requirement was set at 25 kcal/kg CBW/day throughout the ICU stay. CBW equalled actual body weight for participants < 65 years with a BMI < 25 kg/m2 (or if ≥ 65 years, < 30 kg/m2) or adjusted body weight for overweight or obese patients

APACHE, Acute physiology and chronic health evaluation; BMI, body mass index; NUTRIC, Nutrition Risk in Critically ill; RRT, renal replacement therapy; SOFA, sequential organ failure assessment

EN was provided to 118 (99%) patients in tailored nutrition and 116 (98%) in usual care, and PN to 119 (100%) and 17 (14%) patients in tailored nutrition and usual care, respectively.

### Primary outcome

Energy delivered from nutrition sources for the tailored nutrition group was 1796 ± 31 kcal/day versus 1482 ± 32 kcal/day in usual care (mean difference, 313 kcal, 95% CI 226–401 kcal; adjusted mean difference 271 kcal/day, 95% CI 189–354 kcal/day; Fig. 2a, Additional File Fig. 3a). Table 2 shows kcal/kg and proportion of intake variables.

**Fig. 3:**
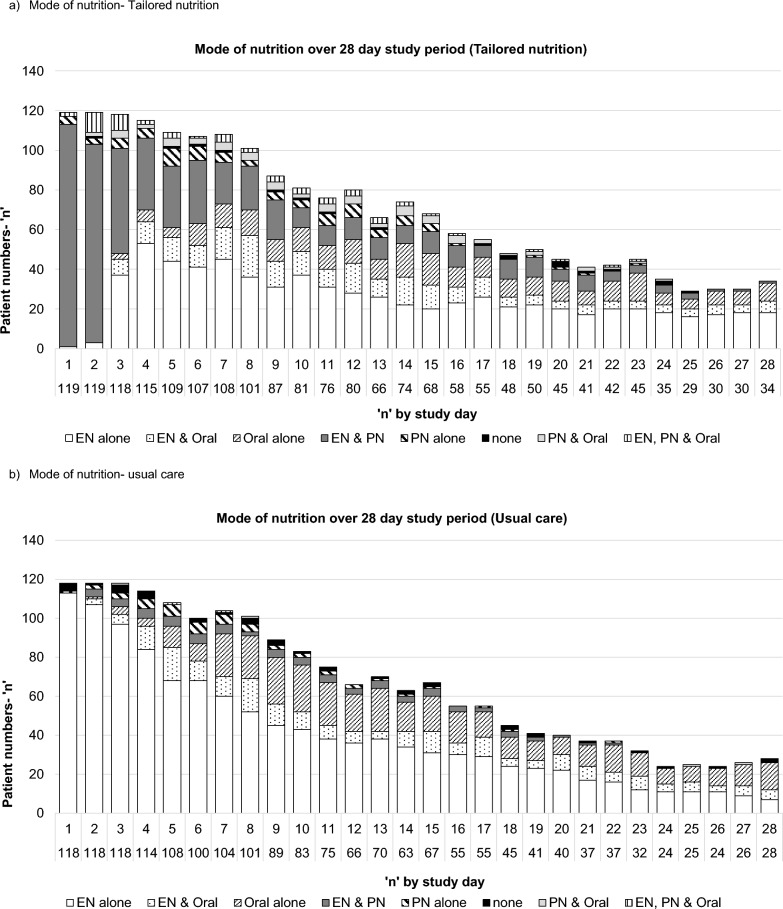
Mode of nutrition provision across 28 day study period. **A** Mode of nutrition- Tailored nutrition, **b** Mode of nutrition- usual care

**Table 2:** Daily nutrition delivery over the 28 day study period and by location

Variable	Tailored nutrition (n = 119)	Usual care (n = 118)	Difference (95% CI)	Adjusted Difference (95% CI)*
*Primary outcome- daily energy provision from EN, PN and oral sources (overall hospital stay)*				
Total energy, kcal/day	1796 ± 31	1482 ± 32	313 (226–401)	271 (189–354)
*Daily energy and protein provision from EN, PN and oral sources (overall hospital stay)*				
Total energy, kcal/kg CBW	21.7 ± 0.3	19 ± 0.3	3.2 (2.2–4.1)	3.4 (2.5–4.3)
Total energy, kcal/kg, actual body weight	20 ± 0.4	17 ± 0.4	2.5 (1.5–3.6)	3.0 (2.2–3.9)
Proportion of study energy requirement provided, %	85 ± 1.3	72 ± 1.3	13.0 (9.4–16.5)	13.7 (10.2–17.2)
Total protein, g	93 ± 1.8	72 ± 1.8	20.9 (15.9–25.9)	18.5 (13.8–23.2)
Total protein, g/kg CBW	1.1 ± 0.02	0.9 ± 0.02	0.22 (0.17–0.27)	0.23 (0.18–0.28)
Total protein, g/kg actual body weight	1.0 ± 0.02	0.8 ± 0.02	0.18 (0.13–0.24)	0.21 (0.16–0.25)
Proportion of protein requirements provided, %	89 ± 2	72 ± 2	16.7 (12.3–21.2)	17.0 (12.4–21.5)
*ICU- Daily energy and protein provision from EN, PN and oral sources*				
Total energy, kcal	1849 ± 34	1576 ± 36	273 (176–369)	221 (129–313)
Total energy, kcal/kg CBW	22 ± 0.4	20 ± 0.4	2.5 (1.5–3.5)	2.8 (1.8–3.8)
Total energy, kcal/kg, actual body weight	20 ± 0.4	18 ± 0.4	1.9 (0.7–3.0)	2.3 (1.4–3.3)
Proportion of study energy requirement provided, %	89 ± 1.4	79 ± 1.5	10.2 (6.2–14.1)	11.0 (7.1–14.9)
Total protein, g	99 ± 1.9	78 ± 2.0	20.8 (15.4–26.3)	17.9 (12.7–23.1)
Total protein, g/kg CBW	1.2 ± 0.02	1.0 ± 0.02	0.21 (0.15–0.27)	0.22 (0.16–0.27)
Total protein, g/kg actual body weight	1.1 ± 0.02	0.9 ± 0.02	0.17 (0.11–0.23)	0.19 (0.14–0.24)
Proportion of protein requirements provided, %	94 ± 2	77 ± 2	16.8 (11.9–21.6)	17.0 (12.0–22.0)
*Ward- Daily energy and protein provision from EN, PN and oral sources*				
Total energy, kcal	1743 ± 41	1388 ± 41	354 (239–469)	322 (211–433)
Total energy, kcal/kg CBW	21 ± 1	17 ± 1	3.8 (2.5–5.1)	4.0 (2.8–5.3)
Total energy, kcal/kg, actual body weight	19 ± 1	16 ± 1	3.1 (1.8–4.5)	3.7 (2.5–4.9)
Proportion of study energy requirement provided, %	80 ± 2	64 ± 2	15.8 (10.8–20.7)	16.5 (11.5–21.4)
Total protein, g	87 ± 2	66 ± 2	21.0 (14.5–27.4)	19.1 (12.9–25.3)
Total protein, g/kg CBW	1.10 ± 0.03	0.80 ± 0.03	0.24 (0.17–0.31)	0.24 (0.18–0.31)
Total protein, g/kg actual body weight	0.95 ± 0.02	0.75 ± 0.03	0.20 (0.13–0.27)	0.23 (0.16–0.29)
Proportion of protein requirements provided, %	84 ± 2	67 ± 2	16.7 (10.8–22.6)	16.9 (10.9–22.9)

Data is presented as n(%), median [IQR] or mean ± SD standard error and mean difference (95%CI)

CBW, calculated body weight; EN, enteral nutrition; PN, parenteral nutrition

*Variables included in the adjusted mean difference Covariate adjustment for; site, age, BMI, APACHE II, frailty and diagnosis

### Secondary outcomes

Protein delivery in tailored nutrition was 93 ± 2 versus 72 ± 2 g/day in usual care (mean difference 21 g/day, 95% CI 16–26 g/day; adjusted mean difference 19 g/day, 95% CI 14–23 g/day; Table 2, Fig. 2b, Additional File Fig. 3b). Mode of nutrition across the study is displayed in Fig. 3 & Additional File Table 2. Additional File Table 2 shows energy and protein delivery from nutrition and non-nutrition sources and Additional File Table 3 by mode over 28 days.

**Table 3:** Clinical outcomes over the 28 day study period

Variable	Tailored nutrition (n = 119)	Usual care (n = 118)	Treatment difference (Intensive vs. Usual) (95%CI)
Central line change during stay	56 (49%)	47 (41%)	RR 1.19 (0.89—1.59)
Blood stream infections (any)	6 (5%)	3 (3%)	RR 1.98 (0.51 – 7.74)
Time to first infection, days	11 [10–15]	9 [5–18]	HR^a^ 1.99 (0.50 – 7.82)
Weight change to hospital discharge, kg	− 4.7 (9.4)	− 5.1 (8.5)	− 0.4 (− 2.8 to 2.0)^+^
Change in weight per day to hospital discharge	− 0.07 (0.56)	− 0.18 (0.54)	− 0.11 (− 0.26 to 0.04)^+^
*ICU*			
Mortality, n (%)	16 (13%)	14 (12%)	RR 1.13 (0.58–2.22)
ICU mobility scale	4 [2–7]	4 [2–6]	0.0 (− 1.6 to 1.6)*
Ventilator free days at D28, days	20 [5–24]	21 [14–25]	− 1.0 (− 5.0 to 3.0)*
*Hospital*			
Mortality, n (%)			
Day 28	18 (15%)	15 (13%)	RR 1.19 (0.63–2.25)
Hospital discharge	20 (17%)	18 (15%)	RR 1.10 (0.61–1.98)

Data is presented as n(%), mean(SD) or median[IQR]. Change in weight per day was calculated by dividing the change in weight between discharge and baseline by the number of days

D28, study day 28; EN, enteral nutrition; ICU, intensive care unit; RR, Relative Risk; HR, Hazard Ratio

^a^Sub-distribution hazard regression model accounting for the competing risk of death, representing the relative probability of infection + Mean difference (95%CI)

*Difference of medians(95%CI)

### Nutrition delivery by location- ICU

ICU length of stay was 10 [6–17] days in tailored nutrition and 8 [5–16] days in usual care. Within ICU, energy delivered from nutrition sources for the tailored nutrition group was 1849 ± 34 kcal/day versus 1576 ± 36 kcal/day in usual care (mean difference, 273 kcal/day, 95% CI 176–369 kcal/day; adjusted mean difference 221 kcal/day, 95% CI 129–313 kcal/day). Protein delivery for tailored nutrition was 99 ± 2 g/day versus 78 ± 2 g/day in usual care (mean difference 21 g/day (95% CI 16–26 g/day); adjusted mean difference 18 g/day (95% CI 14–23 g/day, Table 2, Additional File Table 4). Additional File Fig. 4 displays mode of nutrition delivery in ICU.

### Nutrition delivery by location- ward

Median ward length of stay was 9 [4–17] days in tailored nutrition and 9 [4–18] days in usual care. Energy delivered from nutrition sources for the tailored nutrition group was 1743 ± 41 kcal/day versus 1388 ± 41 kcal/day in usual care (mean difference 354 kcal/day, 95% CI 239–469 kcal/day; adjusted mean difference 322 kcal/day, 95% CI 211–433 kcal/day). Protein delivery from nutrition in tailored nutrition was 87 ± 2 g/day versus 66 ± 2 g/day in usual care (mean difference 21 g, 95% CI 15–27 g/day, adjusted mean difference 19 g/day, 95% CI 13–25 g/day; Table 2, Additional File Table 5). Additional File Fig. 5 displays mode of nutrition delivery on the ward.

### Intervention delivery, clinical and tertiary outcomes

From ICU to hospital discharge, more dietitian reviews were conducted in the tailored nutrition group (median 5 [3–7] vs 4 [2–6] in usual care), and time spent on the intervention was 0.7 [0.5–0.9] hours per occasion or 3 [2–5] hours in total. Additional File 10 provides further data on intervention delivery.

No differences were observed in other clinical secondary or tertiary outcomes (Table 3, Additional File Table 6 & 7, Additional File Figs. 6–10).

### Subgroup analysis

Enhanced energy delivery with tailored nutrition was achieved in all sub-groups (Additional File Fig. 11).

### Adverse Events and protocol deviations:

There were 7 adverse events, all in the tailored nutrition group; 4 (57%) for hyperglycaemia, 1 (14%) for hypertriglyceridemia and 2 (28.5%) other. Of 131 protocol deviations, 20 (15%) were classified as major (12 (5%) ‘randomised but not eligible’ and 8 (3%) ‘patient received more than 120% of energy requirements’) (Additional File 9).

## Discussion

In this multicentre RCT a tailored nutrition intervention commenced in the acute late phase of critical illness within ICU and continued through to hospital discharge was investigated. The intervention demonstrated a significant increase in energy and protein delivery, utilising supplemental PN in the ICU and oral nutrition supplements in the late ICU and post-ICU period. No differences in secondary or tertiary clinical outcomes were observed.

Within ICU, we achieved an increase in energy provision with the addition of PN to supplement EN, a proven strategy to enhance energy delivery in ICU [25–27]. The use of supplemental PN has been considered controversial since the publication of a large RCT suggested harm in patients who received early supplemental PN (day 3) compared to those who commenced late (day 8) [3]. However, other large RCTs and meta-analyses conducted since have shown neither harm nor benefit from PN, either provided alone or supplemental [7, 25–29]. In the ICU, our intervention met nearly 90% of the estimated study requirement. This is comparable to other studies using supplemental PN in the initial 7–10 days and to the TARGET trial which tested the only augmented EN strategy within ICU [4, 25, 27].

On the ward, our intervention increased energy provision by 322 kcal/day (to 80% adequacy compared to 64% in usual care) primarily through oral nutrition, potentially reducing reliance on artificial nutrition in later stages of illness. This intervention, spanning a median of 19 days, represents the longest duration of nutrition therapy ever reported in a trial of nutrition during critical illness. Unlike trials focused only on the ICU period, the overall energy separation achieved was not as pronounced, possibly due to the longer study duration or the high standard of usual care provided [4]. Notably, there has been only one other trial in acute care investigating nutrition a intervention for the entire hospitalisation period, showing reduced mortality and fewer adverse outcomes with a tailored oral nutrition approach in acutely unwell, non-critically ill medical inpatients from Switzerland [14].

Tailored nutrition provision required substantial additional resources for nutrition care compared to typical practices in ANZ and internationally. Previous observational study designs have shown that the presence of a dietitian within ICU can enhance nutrition delivery and increase team focus on nutrition but there is limited guidance on staffing resources for dietitians in ICU or acute hospitalisation [30, 31]. Furthermore, observational data from the late ICU and post-ICU period describing usual care nutrition process describes very low nutrition provision when minimal resources for nutrition care are available, increasing when a higher level of nutrition care is provided [16, 20]. To address this, our late ICU and post-ICU intervention involved a dietitian-delivered, tailored nutrition plan with three weekly formal reviews, individualised changes to hospital food based on preferences, daily study oral nutrition supplements, and additional hospital supplements as needed. Our study, and the largest study conducted outside of the critical care setting, shows that tailored nutrition consisting of oral nutrition enhancement is successful with an individual approach; however the resource, cost and clinical implications of this remains to be determined in the ICU and post-ICU setting [14].

The impact of our intervention on important patient centred outcomes remains unclear due to insufficient power to detect clinical differences. Trials investigating *early* increased energy delivery have not shown benefit and two recent trials investigating increased protein delivery also found no benefit [6, 32]. The first found a signal for harm with early protein delivery in a subgroup of patients with baseline renal dysfunction and the second indicated long-term harm on quality of life to 180 days with increased protein delivery commenced in the acute late phase of illness [6, 32]. In addition, important physiological work published during the conduct of our trial indicated that protein utilisation for muscle protein synthesis is blunted compared to healthy controls [2]. Our study differs from previous trials in the delayed initiation (commencing from day 3–5 in the post-acute phase) and prolonged duration which intuitively could result in an improved response to nutrition. Future research should address the impact of longer-term nutrition interventions on critical patient-centred outcomes, such as quality of life and function, through adequately powered RCTs. Other key evidence gaps include determining the optimal timing, dose and mechanistic aspects of nutrition interventions. This study also suggests that individualised oral nutrition interventions in the later stages of critical illness can enhance energy and protein delivery, highlighting a key area for future investigation.

This study has many strengths, including that the intervention commenced in the acute late phase of critical illness and continued until hospital discharge, is multicentre in design (including major metropolitan and smaller regional centres) and adhered to rigorous trial management processes. Limitations include the unblinded trial design, potentially leading to higher nutrition provision in the usual care group. While the high standard of care is reflective of care in Australia and New Zealand, it may limit generalisability of results to countries with a lower standard of care. We used data from a previously conducted trial within out group to determine our sample size; however, the minimally important difference for energy delivery in critical illness is speculative. Despite this, we achieved higher energy separation than anticipated and provision was comparable to a large double-blinded RCT of EN in Australia and New Zealand [4]. We commenced the intervention between day 3–5 of ICU admission, with the aim to avoid the acute early phase of illness; however, this is a theoretical timepoint and there is potential that some patients were still within the acute early phase of illness. The use of the Ur/CR ratio shows promise as a marker of a patients’ ability to process nutrition according to clinical state. Other potential markers, such as IL6 and CRP, are not routinely monitored in Australia and New Zealand and were not collected [33]. Moreover, their use as biomarkers to direct nutrition care has not been confirmed and should be a focus of future work. Despite this, this trial is one of few with interventions to commence in the acute-late phase of critical illness. It is plausible that the response to nutrition is influenced by sex. Our trial included more patients of male sex, which is a known limitation in critical care research generally; however, this does limit conclusions for patients of female sex [34]. To maintain pragmatism, data collection on the post-ICU ward was reduced to three times per week, resulting in some unavailable information. Similarly, information pertaining to SOFA scores was not collected daily and in the absence of detailed timing pertaining to surgical intervention, may reflect perioperative inflammation. The COVID-19 pandemic significantly impacted the trial, causing recruitment pauses (12–23 active sites during recruitment), missed patients, resource constraints, and potential practice changes, the true impact of which is unquantifiable. Despite no observed differences in clinical or secondary outcomes, this study was not powered to detect clinical differences and no adjustment for multiple comparisons was performed.

## Conclusion

This study demonstrates that a tailored nutrition intervention commenced in the acute late period of critical illness within ICU and continued until hospital discharge, achieved a significant increase in daily energy delivery for patients initially admitted to the ICU. The clinical implications of this remain to be determined.

## Supplementary Information

Additional file1 (PDF 1444 KB)

## Data Availability

Data requests will be considered on an individual basis and should be made in writing to the corresponding author.
