# Supplementary material for: The impact of a tailored nutrition intervention delivered for the duration of hospitalisation on daily energy delivery for patients with critical illness (INTENT): a phase II randomised controlled trial
Source: Crit Care. 2025 Jan 6;29:8. doi: 10.1186/s13054-024-05189-3 (PMC11706088; doi:10.1186/s13054-024-05189-3)
Supplement: Supplementary file 1 — Additional file1 (PDF 1444 KB) [file 13054_2024_5189_MOESM1_ESM.pdf]

THE IMPACT OF A TAILORED NUTRITION INTERVENTION DELIVERED FOR THE DURATION OF HOSPITALISATION ON DAILY ENERGY DELIVERY FOR PATIENTS WITH CRITICAL ILLNESS (INTENT); A PHASE II RANDOMISED CONTROLLED TRIAL

ADDITIONAL FILE

Authors: Emma J Ridley[1,2], Michael Bailey[1,], Marianne J Chapman[3,4], Lee-anne S Chapple[3,4], Adam M Deane[5], Marlene Gojanovic[1], Alisa M Higgins[1], Carol L Hodgson[1,6,7,8], Victoria L King[1], Andrea P Marshall[9,10], Eliza G Miller[1], Shay P McGuinness[1, 11], Rachael L Parke[1,11,12], Eldho Paul [1], Andrew A Udy[1,6] and the Australian and New Zealand Intensive Care Society Clinical Trials Group

Authors email addresses: Emma.Ridley@monash.edu; Michael.Bailey@monash.edu; Marianne.Chapman@sa.gov.au; Lee-anne.Chapple@adelaide.edu.au; Adam.Deane@mh.org.au; Marlene.Gojanovic@monash.edu; Lisa.Higgins@monash.edu; Carol.Hodgson@monash.edu; Victoria.King@monash.edu; Andrea.Marshall@health.qld.gov.au; Eliza.Miller@novotech-cro.com; ShayMc@adhb.govt.nz; RParke@adhb.govt.nz; Andrew.udy@monash.edu

Corresponding author: A/Prof Emma J Ridley; emma.ridley@monash.edu; Phone: +61 3 9903 0350 Australian and New Zealand Intensive Care Research Centre, Department of Epidemiology and Preventive Medicine, Monash University. Postal address: 553 St Kilda Road, Melbourne, Victoria, Australia.

Affiliations:

1 Australian and New Zealand Intensive Care Research Centre, School of Public Health and Preventive Medicine, Department of Epidemiology and Preventative Medicine, Monash University, Melbourne, Victoria, Australia

2 Nutrition Department, The Alfred Hospital, Melbourne, Victoria, Australia

3 Adelaide Medical School, University of Adelaide, Adelaide, South Australia, Australia

4 Intensive Care Unit, Royal Adelaide Hospital, Adelaide, South Australia, Australia

5 Department of Critical Care, Melbourne Medical School, The University of Melbourne, Australia

6 Intensive Care Unit, Alfred Hospital, Melbourne, Victoria, Australia

7 Department of Critical Care, The George Institute for Global Health, Sydney, NSW, Australia

8 Department of Critical Care, The University of Melbourne, Melbourne, Victoria, Australia

9 Gold Coast Hospital and Health Service, Southport, Queensland, Australia

10 School of Nursing and Midwifery, Griffith University, Gold Coast Campus, Southport, Queensland, Australia

11 Cardiothoracic and Vascular Intensive Care Unit, Auckland City Hospital, Auckland, New Zealand

12 School of Nursing, The University of Auckland, Auckland, New Zealand

ADDITONAL FILE CONTENTS

Additional file 1. Inclusion and exclusion criteria 5

Additional file 2. INTENT investigators, participating sites, DSMC membership and site recruitement 6

Additional file 3. Ethics approval and consent process 9

Additional file 4. Product information for Olimel N12E with electrolytes and additions 10

Additional file 5. Nutrition information for Fortisip Compact Protein and Forticreme Complete 11

Additional file 6. INTENT study energy requirement table 12

Additional file 7. energy requirement calculation and strategies to minimise overfeeding 13

Additional file 8. Definitions for outcome variables and statistical analysis details 15

Additional file 9. Adverse events and protocol deviations 17

Additional file 10. Data on intervention characteristics and delivery 21

ADDITIONAL TABLES 22

ADDITIONAL TABLE 1. Further baseline participant characteristics 22

ADDITIONAL TABLE 2. Energy delivery from nutrition and non-nutrition sources over 28 days and by location 24

ADDITIONAL TABLE 3. Energy and protein provision from each mode of nutrition over 28 days 25

ADDITIONAL TABLE 4. Daily energy and protein delivery in ICU according to source 27

ADDITIONAL TABLE 5. Daily energy and protein delivery on the ward according to source* 29

ADDITIONAL TABLE 6. Daily clinical information in ICU 31

ADDITIONAL TABLE 7. Outcome data 32

ADDITIONAL FIGURES 33

ADDITIONAL FIGURE 1. Full study process 33

ADDITIONAL FIGURE 2. Management of interventional PN in the intensive nutrition care arm 34

ADDITIONAL FIGURE 3. Delivery of nutrition over 28 day study period 35

ADDITIONALFIGURE 4. Mode of nutrition delivery in ICU by day 37

ADDITIONAL FIGURE 5. Mode of nutrition delivery on the ward 39

ADDITIONAL FIGURE 6. Cumulative incidence plots 41

ADDITIONAL FIGURE 7. PATIENT SURVIVAL IN DAYS FROM RANDOMISATION (CENSORED AT 250 DAYS) 43

ADDITIONAL FIGURE 8. Clinical frailty score 43

ADDITIONAL FIGURE 9. Patient weight 44

ADDITIONAL FIGURE 10. Patient status across the 28 day study period 44

ADDITIONAL FIGURE 11. Daily energy difference (kcal) for specified sub-groups 45

ADDITIONAL FILE 1. INCLUSION AND EXCLUSION CRITERIA

Inclusion criteria

Patients in intensive care were eligible for enrolment if they met all of the following inclusion criteria:

1. Admitted to any intensive care unit for between 72 and 120 hours
2. Receiving invasive ventilator support
3. At least 18 years of age
4. Had central venous access suitable for PN solution administration
5. Had one or more organ system failure (respiratory, cardiovascular or renal) related to their acute illness defined as:

a) $PaO_2/FiO_2 \leq 300$ mmHg

b) Receiving one or more continuous inotrope/vasopressor infusion started within the previous 4 hours at a minimum dose of:
   - Noradrenaline ≥ 0.1mcg/kg/min
   - Adrenaline ≥ 0.1 mcg/kg/min
   - Any dose of vasopressin
   - Milrinone > 0.1 mcg/kg/min

c) Renal dysfunction defined as:
   - Serum creatinine 2.0-2.9 times baseline or
   - Urine output 0.5ml/kg/hr for ≥ 12 hours or
   - Currently receiving renal replacement therapy

d) An intracranial pressure monitor or ventricular drain in situ

Exclusion criteria

Patients were excluded if they met one or more of the following exclusion criteria:

1. Both EN and PN could not be delivered at enrolment
2. PN was already being received
3. Clinician believed a specific parenteral formula was indicated
4. Death was imminent in the next 96 hours or there was a current treatment limitation in place or the patient was unlikely to survive to 180-days due to underlying/chronic illness
5. More than 80% of energy requirements had been satisfactorily delivered via the enteral route in the last 24 hours
6. Dialysis dependent chronic renal failure
7. Suspected or known pregnancy
8. Product was contraindicated
9. The treating clinician did not believe the study to be in the best interest of the patient

ADDITIONAL FILE 2. INTENT INVESTIGATORS, PARTICIPATING SITES, DSMC MEMBERSHIP AND SITE RECRUITEMENT

Trial Investigators: A/Prof Emma Ridley (Chief Investigator), Prof Michael Bailey, Prof Marianne Chapman, A/Prof Lee-anne Chapple, Prof Adam Deane, Prof Carol Hodgson, Ms Victoria King, Prof Andrea Marshall, Dr Shay McGuinness, A/Prof Rachael Parke, Prof Andrew Udy

Statisticians: Prof Michael Bailey, Dr Eldho Paul

Health Economist: Dr Lisa Higgins

Project Management: Marlene Gojanovic, Victoria King, Eliza Miller

Independent Data and Safety Monitoring Committee: Dr Rob Fowler (Chair), Prof David Harrison, Prof Paul Wischmeyer

Participating Sites (principal investigators and key contributors alphabetically by institution and all in Australia unless specified):

Study sites were identified via an expression of interest process through the Australian and New Zealand Intensive Care Society Clinical Trials Group (ANZICS CTG).

Auckland City Hospital Cardiothoracic and Vascular Intensive Care Unit, Auckland, NZ, Farisha Ali, Rebecca Baskett, Magdalena Butler, Keri-Anne Cowdrey, Eileen Gilder, Lydia Gillan, Su-Zahn Koorts, Brittany Mason, Ellie McMahon, Shay McGuinness, Karina O'Connor, Rachael Parke, Melissa Robertson, Samantha Ryan, Anna Small, Andrew Xia; Austin Hospital, Melbourne, VIC, Megan Berner, Christine Choong, Glenn Eastwood, Kate Hamilton, Daryl Jones, Leah Peck, Helen Young; Bendigo Health, Bendigo, VIC, Lauren Ballantyne, Catherine Boschert, Cameron Knott, Bridget Roberts, Julie Smith; Blacktown Hospital, Blacktown, NSW, Zoe Flick, Kalpesh Gandhi, Barbara Hannah, Yvonne Li, Kiran Nand, Treena Sara, Sylvia Wei; Box Hill Hospital, Melbourne, VIC, Lina Briek, Graeme Duke, Kym Gellie, Stephanie Hunter, Nicole Robertson; Epworth Hospital Richmond, Melbourne, VIC, Jonathan Barrett, Brydie Cleeve, Caroline Guille, Gabrielle Hanlon, Sarah Jelly-Butterworth, Julie O'Donnell, Carmel Zoanetti; Frankston Hospital, Frankston, VIC, Zhoe Coram, Karen Edis, Mike Gomez, Alice Goodman, Kavi Haji, Jenny Moss, Jodie Prendergast, Janet Tam, Ravindranath Tiruvoipati, Fiona Turnbull; Gold Coast University Hospital, Southport, QLD, Julie Cussen, Maimoonbe

Gough, Sarah Lovelock, Lisa Mahoney, Andrea Marshall, David Pearson, Mandy Tallott; Grampians Health Ballarat – Acute site, Ballarat, VIC, Emily Ainslie, Kate Flynn, Kerri Gordon, Tim Stewart, Larissa Telfer, Victoria Williams; Lyell McEwin Hospital, Adelaide, SA, Vishwanath Biradar, Hanaa Chahine, Edda Jessen, Matia Kapsambelis, Melissa Lydyard, Ashlee Martin, Julie Puccini, Natalie Soar, Leah Sommerfield; Mater Misericordiae Ltd, Brisbane, QLD, Bronwyn Bartholomew, Peter Collins, Claire Filet, Mackenzie Finnis, Chloe Jobber, Katherine Jongebloed, Isabel Anne Leditschke; Middlemore Hospital, Auckland, NZ, Kimberley Browning, Jennifer Chang, Dinu Girijadevi, Areege Hussein, Vivian Lai, Rima Song, Tony Williams; Monash Medical Centre, Melbourne, VIC, Oshara de Silva, Ashlee Gervasoni, Carolyn Hall, Lauren Hanna, Sheree Phillips, Yahya Shehabi; Nepean Hospital, Sydney, NSW, Rebecca Gresham, Matin Jamei, Sheeja Joy, Julie Lowrey, Kristy Masters, Ian Seppelt, Wendy Tu, Christina Whitehead; Northern Hospital, Melbourne, VIC, Tina Aboltins, Hayley Collins, Rachael Evans, Angaj Ghosh, Simone Said, Vivian Tsang; Princess Alexandra Hospital, Brisbane, QLD, Lynette De Groot, Ra'eesa Doola, Meg Harward, Cassie Jones, Josephine Mackay, Jason Meyer, Tahnie Takefala, James Walsham; Redcliffe Hospital, Redcliffe, QLD, Stuart Baker, Anthony Khoo, Shannon Lewis, Alyce Nissen, Alexis Tabah, Alicia Wiese; Royal Darwin Hospital, Darwin, NT, Lewis Campbell, Miriam Chin, Rebecca Garcia, Kirsty Smyth, Annabel Thallon; Royal Melbourne Hospital, Melbourne, VIC, Emma Bidgood, Jessica Browne, Kathleen Byrne, Adam Deane, Kate Fetterplace, Hilda Griffin, Sarah Phillips, Kym Wittholz; The Alfred, Melbourne, VIC, Jasmin Board, Peta Bretag, Aidan Burrell, Adam Cunningham, Dashiell Gantner, Ramez Hanna, Kate Lambell, Karina Lay, Elisa Licari, Lee Lin Loh, Emma Martin, Phoebe McCracken, Jenna Obeid, Caitlin Rabel, Peter Thanhauser, Andrew Udy, Chloe Vadiveloo, Cyndi Wong, Meredith Young; The Prince Charles Hospital, Brisbane, QLD, Cameron French, Greta Hollis, Adrian Powlesland, Kiran Shekar, Marion Vasudevan, Emma Whitmore; The Queen Elizabeth Hospital, Adelaide, SA, Tennealle Direen, Martine Hatzi, Cathy Kurenda, Sandra Peake, Amber Thatcher, Patricia Williams; University Hospital Geelong, Geelong, VIC, Michelle Horton, Nima Kakho, Matthew Maiden, Tania Salerno, Jemma Trickey.

Recruitment by participating site:

	2018	2019	2020	2021	2022	2023	TOTAL
Number of active sites during the year	1	12	12-15	23	16	16	
Number of paused sites during the year*	0	0	6-8	1-10	0	0	
Site Number	Number of Participants Recruited						
1	0	10	17	9	16	2	54
2	4	19	7	9	8	2	49
3	0	0	0	12	16	1	29
4	0	0	0	11	6	0	17
5	0	3	3	4	1	1	12
6	0	1	3	3	2	1	10
7	0	0	0	5	4	0	9
8	0	2	1	2	2	0	7
9	0	3	2	1	1	0	7
10	0	2	3	2	0	0	7
11	0	2	2	0	2	0	6
12	0	0	1	3	1	0	5
13	0	0	0	3	2	0	5
14	0	0	0	3	2	0	5
15	0	4	0	0	0	0	4
16	0	3	1	0	0	0	4
17	0	0	0	3	0	0	3
18	0	0	0	3	0	0	3
19	0	0	0	1	0	0	1
20	0	0	0	0	1	0	1
21	0	1	0	0	0	0	1
22	0	0	1	0	0	0	1
23	0	0	0	0	0	0	0
**TOTAL**	**4**	**50**	**41**	**74**	**64**	**7**	**240**

* Sites needed to pause for various durations depending on waves of the COVID-19 pandemic. Some were not able to commence again due to staffing capacity, reflected in lower number of active sites in 2022/23.

ADDITIONAL FILE 3. ETHICS APPROVAL AND CONSENT PROCESS

Ethics approval was obtained from the Alfred Hospital Ethics Committee (HREC/18/Alfred/101) and the Human Research Ethics Committee of the Northern Territory Department of Health (2019-3372) in Australia and the New Zealand Central Health and Disability Ethics Committee (18/NTA/222/AM01) in New Zealand. The original protocol was approved on 31st July 2018, with a subsequent minor protocol amendment of editorial changes for clarity approved on 8th January 2020 (detailed in the protocol publication [1]).

Patients were unable to provide informed consent prior to randomisation/enrolment. Accordingly, in Australia, the patient's medical treatment decision maker (relative/friend) or legal surrogate was approached to provide consent for the patient to participate prior to enrolment in the study. In New Zealand, the respective ethics committee approved the use of a deferred consent model. Family/Whanau were approached as soon as possible to inform them about study enrolment and to seek their views on whether or not the patient would be agreeable to being included in the research study. In both countries, the patient was approached to give consent for continued participation in the trial if they recovered the ability to do so and the timing was appropriate.

ADDITIONAL FILE 4. PRODUCT INFORMATION FOR OLIMEL N12E WITH ELECTROLYTES AND ADDITIONS

Contents	Compounded Ready To Use Parenteral Nutrition (per 1000ml bag)
Total nitrogen (g)	12.0
Amino acid (g)	75.9
Glucose (g)	73.3
Lipid as ClinOleic (g)	35.0
Total energy (kcal)	950
Non protein energy (kcal)	640
Glucose energy (kcal)	290
Lipid energy (kcal)	350
Sodium (mmol)	35
Potassium (mmol)	30
Magnesium (mmol)	4.0
Calcium (mmol)	3.5
Phosphate (including lipid) (mmol)	15.0
Acetate (mmol)	70
Chloride (mmol)	45
Osmolarity (mOsm/L)	1270
**Additions per bag of PN**	
Baxter's Multiple Trace Elements with Iron (mcg)	Per ml (10ml is added to each 1L PN bag)
Zinc	650
Copper	51.5
Manganese	5.5
Chromium	1.0
Selenium	8.0
Iodide	13
Molybdenum	1.9
Iron	110
Ascorbate (Vitamin C) for stability (mg per bag)*	120
Cernevit (ml per bag)	5

* Sodium Ascorbate in Australia, Ascorbate acid in NZ.

ADDITIONAL FILE 5. NUTRITION INFORMATION FOR FORTISIP COMPACT PROTEIN AND FORTICREME COMPLETE

Manufactured by Nutricia Australia Pty Limited*

	**Fortisip Compact Protein (per 125 ml bottle)**	**Forticreme Complete (per 125 g pot)**
Energy (kcal)	300	200
Energy (kJ)	1263	844
Protein (g)	18	11.9
Casein (g)	16.8	9.4
Whey (g)	1.3	2.6
Carbohydrates (g)	30.5	24
Sugars (g)	16.6	13.3
Lactose (g)	0.38	0.13
Fat (g)	11.8	6.3
Saturates (g)	1.1	0.9
Monounsaturates (g)	7.1	3.8
Polyunsaturates (g)	3.5	1.6
Omega 6:Omega 3	5.1:1	5.1:1
Fibre (g)	0	0.13
Water (ml)	78.8	80
Sodium (mg)	50	78.8
Sodium (mmol)	2.1	3.4
Potassium (mg)	131	231
Potassium (mmol)	3.4	5.9
Calcium (mg)	43.8	163
Phosphorous (mg)	375	141
Magnesium (mg)	68.8	25
Chloride (mg)	75	80
Ca:P	1.2:1	1.1:1
Vitamin A (μg-RE)	325	256
Vitamin D (μg)	2.6	2.1
Vitamin E (mg α-TE)	4.6	3.4
Vitamin K (μg)	20	16.3
Vitamin C (mg)	37.5	20
Thiamin (mg)	0.56	0.46
Riboflavin (mg)	0.6	0.5
Niacin (mg NE)	4.5	3.5
Vitamin B6 (mg)	0.66	0.52
Vitamin B12 (μg)	1.4	0.64
Folic Acid (μg)	100	80
Pantothenic Acid (mg)	2	1.6
Biotin (μg)	15	8
Iron (mg)	2.6	3.3
Zinc (mg)	3	2
Manganese (mg)	0.79	0.8
Copper (μg)	438	540
Iodine (μg)	57.5	42.5
Molybdenum (μg)	25	22.5
Selenium (μg)	17.5	13.8
Chromium (μg)	16.3	13.8
Fluoride (mg)	0.24	0.23
Choline (mg)	138	110
Osmolality (mOsmol/kg$H_2$0)	900	820

Mmol, millimole; μg, microgram; μg-RE, μg retinol equivalents; mg α-TE, milligram alpha- tocopherol equivalents; mg NE, milligram niacinamide; mOsmol/kgH20, milliosmols (one-thousandth of an osmole) per kilogram of water (mOsmol/kg)

* Please note the ingredients list and nutritional information is representative of the Vanilla flavour only for both products. There are minor variations between different flavours.

ADDITIONAL FILE 6. INTENT STUDY ENERGY REQUIREMENT TABLE

Patients calculated body weight (kg)	Daily requirement at 25 kcal/kg	Patients calculated body weight (kg)	Daily requirement at 25 kcal/kg	Patients calculated body weight (kg)	Daily requirement at 25 kcal/kg
40	1000	77	1925	114	2850
41	1025	78	1950	115	2875
42	1050	79	1975	116	2900
43	1075	80	2000	117	2925
44	1100	81	2025	118	2950
45	1125	82	2050	119	2975
46	1150	83	2075	120	3000
47	1175	84	2100	121	3025
48	1200	85	2125	122	3050
49	1225	86	2150	123	3075
50	1250	87	2175	124	3100
51	1275	88	2200	125	3125
52	1300	89	2225	126	3150
53	1325	90	2250	127	3175
54	1350	91	2275	128	3200
55	1375	92	2300	129	3225
56	1400	93	2325	130	3250
57	1425	94	2350	131	3275
58	1450	95	2375	132	3300
59	1475	96	2400	133	3325
60	1500	97	2425	134	3350
61	1525	98	2450	135	3375
62	1550	99	2475	136	3400
63	1575	100	2500	137	3425
64	1600	101	2525	138	3450
65	1625	102	2550	139	3475
66	1650	103	2575	140	3500
67	1675	104	2600	141	3525
68	1700	105	2625	142	3550
69	1725	106	2650	143	3575
70	1750	107	2675	144	3600
71	1775	108	2700	145	3625
72	1800	109	2725	146	3650
73	1825	110	2750	147	3675
74	1850	111	2775	148	3700
75	1875	112	2800	149	3725
76	1900	113	2825	150	3750

ADDITIONAL FILE 7. ENERGY REQUIREMENT CALCULATION AND STRATEGIES TO MINIMISE OVERFEEDING

Body weight and height determination:

- Actual body weight was used if recorded in the past 6 weeks. Otherwise, the dietitian's estimate was preferred or an estimate by the research team.
- Actual height was used if known; Otherwise, it was calculated using demi-arm span as preferred or estimated by the research team.

Calculated body weight determination and corresponding energy requirements

Calculated body weight (CBW) in kg was used for the calculation of nutritional targets in the ICU, defined as follows;

For patients under 65 years of age:

a) The patient's actual weight if their body mass index (BMI) was deemed to be <25kg/m^2

b) BMI ≥ 25 kg/m^2 ≤ 40 kg/m^2 : CBW was set at 'actual weight + ideal weight/2' where 'ideal weight' was equivalent to a BMI of 23 kg/m^2

c) BMI > 40 kg/m^2: CBW was set at 'actual weight + ideal weight/2' where 'ideal weight' was equivalent to a BMI of 25 kg/m^2

For patients 65 years of age and over:

a) The patient's actual weight if their BMI was deemed to be < 30kg/m^2

b) BMI ≥30 kg/m^2: CBW was set at 'actual weight + ideal weight/2' where 'ideal weight' was a weight equivalent to a BMI of 27 kg/m^2

The resulting study energy requirements for a given CBW are provided in the table at Additional File 6.

Safety features of the intervention to minimise the risk of overfeeding included:

- Energy requirements were set using an adjusted body weight for participants who were overweight or obese
- All energy sources (EN, propofol, glucose (>25%), any oral nutrition, and PN delivered during any EN interruption(s)) were included when determining the daily need for interventional PN following randomisation
- The maximum amount of energy provided by the interventional PN was 20 kcal/kg CBW/day or equivalent to 80% of the study energy requirement

- EN rates were revised to ensure 80-100% of the participants' study energy requirement was provided where propofol and EN collectively provided >110% of the participants study energy

ADDITIONAL FILE 8. DEFINITIONS FOR OUTCOME VARIABLES AND STATISTICAL ANALYSIS DETAILS

Duration of invasive ventilation, time to blood stream infection, ICU length of stay and Hospital length of stay were measured in days from randomization and were censored at 28, 28,90 and 180 days respectively. Ventilator Free Days (VFDs) until day 28 represents the number of full days in which a patient did not receive ventilation during the first 28 days. All patients that died within 28 days were assigned a score of 0. For increased transparency, all duration variables are additionally reported separately for survivors and non-survivors.

Patient survival was analysed using Cox-proportional hazards regression including clustering for site with results reported as Hazard ratios (95% CI) and presented as Kaplan Meier survival curves. Model assumptions for time to event analyses were assessed through the analysis of the Schoenfeld residuals against time.

Frailty and weight were analysed using hierarchical mixed linear modelling with patients nested within sites and both patients and sites treated as random effects, fitting main effect for treatment and time, and an interaction between the later to determine if treatment behaved differently over time, with results reported as least square means ± standard errors and mean differences (95% CI). VFDs and ICU mobility at discharge were compared between groups using quantile regression with results reported as median [IQR] and difference of medians (95% CI).

Subgroup analysis was performed for the primary outcome on seven subgroups determined at baseline:

- Sex
- Age <=60 vs Age>60
- APACHE II <=16 vs >16
- BMI<=30 vs >30
- Clinical Frailty Score <=3 vs >3
- Patients receiving renal replacement therapy Yes vs No
- Patients admitted for cardiac surgery Yes vs No

Heterogeneity between subgroups was determined by fitting main effects for treatment, subgroup and an interaction between treatment and subgroup, with results reported as forest plots.

Central Line Associated Blood Stream Infection (CLABSI) was added as a tertiary outcome of interest post-hoc. In Australia, CLABSI is defined as “a laboratory-confirmed bloodstream infection in a patient where the central line was in place for > 2 calendar days (48 hours)* on the date of the event, with day of device placement being Day 1” [2]. In New Zealand, central line associated bacteraemia (CLAB) is defined as a significant bloodstream infection that occurs in a patient who has a central line in place OR in a patient who has had a central line removed within 48 hours with no other apparent focus on infection [3].

ADDITIONAL FILE 9. ADVERSE EVENTS AND PROTOCOL DEVIATIONS

Adverse events and serious adverse events:

Adverse events (AEs) are defined as any untoward medical occurrence in a patient or clinical investigation subject administered an investigational intervention. Any such event does not necessarily have to have a causal relationship with this treatment (adapted from the Note for Guidance on Clinical Safety Data Management: Definitions and Standards for Expedited Reporting (CPMP/ICH/377/95 July 2000).

It is recognised that the patient population with critical illness will experience a number of common aberrations in laboratory values, signs and symptoms due to the severity of the underlying illness and the impact of standard therapies. These did not necessarily constitute an AE unless they require significant intervention or were considered to be of concern in the investigator's clinical judgement.

AEs were collected from randomisation until hospital discharge. The AE was followed up until the event was resolved or explained (to day 180 post-randomisation). Frequency to follow-up was left to the discretion of the investigator. AEs will be reported in the eCRF.

Serious Adverse Events (SAEs) are defined in accordance with the Note for Guidance on Clinical Safety Data Management: Definitions and Standards for Expedited Reporting (CPMP/ICH/377/95) (July 2000) as any untoward medical occurrence that:

- Results in death
- Is life-threatening
- Requires inpatient hospitalisation or prolongation of existing hospitalisation
- Results in persistent or significant disability/incapacity
- Is a congenital anomaly/birth defect
- Is an important medical event which may require intervention to prevent one of the previously listed outcomes

Events which were part of the participants natural history of the primary disease process, or which were expected complications of critical illness (including death), were not reported as SAEs. This practice is consistent with recommendations specific to adverse event reporting in trials including critically ill participants [4]. All SAEs considered to be potentially causally related to the study intervention or were

of concern in the investigator's judgment were reported to Baxter Healthcare Corporation (the funding body), the respective ethics committee and the DSMC.

Protocol deviations:

Pre-specified protocol deviations were categorised into major and minor (Table 2). Major protocol deviations included: (1) patients randomised but deemed ineligible; and (2) delivery of the incorrect rate of PN resulting in greater than 120% of a participants' energy requirements met.

Protocol deviations – Per patient

**Reasons for PD as reported by investigators**	**Tailored nutrition (n=119)**	**Usual care (n=118)**
**Major deviations**		
Patient received energy >120%	14 (12%)	4 (3%)
Patient randomised but not eligible	4 (3%)	9 (8%)
**Minor deviations**		
Incorrect rate of study PN administered*	76 (64%)	4 (3%)
Patient randomised but study PN not administered	38 (32%)	0 (0%)
Dispensing/dosing error	21 (18%)	1 (1%)
Study PN not commenced within 2 hours of randomisation on day 1	15 (13%)	0 (0%)
Unapproved procedure	14 (12%)	7 (6%)
Non-study PN administered when study PN should have been administered	12 (10%)	6 (5%)
Study oral supplement not prescribed when indicated	11 (9%)	0 (0%)
Study energy requirement not targeted	9 (8%)	8 (7%)
Did not receive study PN on the day of randomisation	0 (0%)	0 (0%)
Other	6 (5%)	5 (4%)

Values indicated with N are number of patients.
Abbreviations: PD protocol deviation.

Protocol deviations – Events

Reasons of PD as reported by investigators	Tailored nutrition (n=119)	Usual care (n=118)
**Major deviations**		
Patient received energy >120%	15 (3%)	10 (9%)
Patient randomised but not eligible	4 (1%)	9 (8%)
**Minor deviations**		
Incorrect rate of study PN administered*	210 (44%)	5 (5%)
Patient randomised but study PN not administered	95 (20%)	0 (0%)
Study energy requirement not targeted	35 (7%)	39 (35%)
Dispensing/dosing error	30 (6%)	1 (1%)
Unapproved procedure	27 (6%)	9 (8%)
Non-study PN administered when study PN should have been administered	25 (5%)	39 (35%)
Study oral supplement not prescribed when indicated	18 (4%)	0 (0%)
Study PN not commenced within 2 hours of randomisation on day 1	15 (3%)	0 (0%)
Did not receive study PN on the day of randomisation	0 (0%)	0 (0%)
Other	15 (3%)	9 (8%)

Values indicated with N are number of events.
Abbreviations: PD protocol deviation.

<u>* Incorrect rate of study PN administered:</u>

76 patients had 210 occurrences of incorrect PN rate (145 (67%) missed or withheld PN, median (IQR) 2 [1-3] events per person).

Most Incorrect PN rate occurred on days 2 & 3

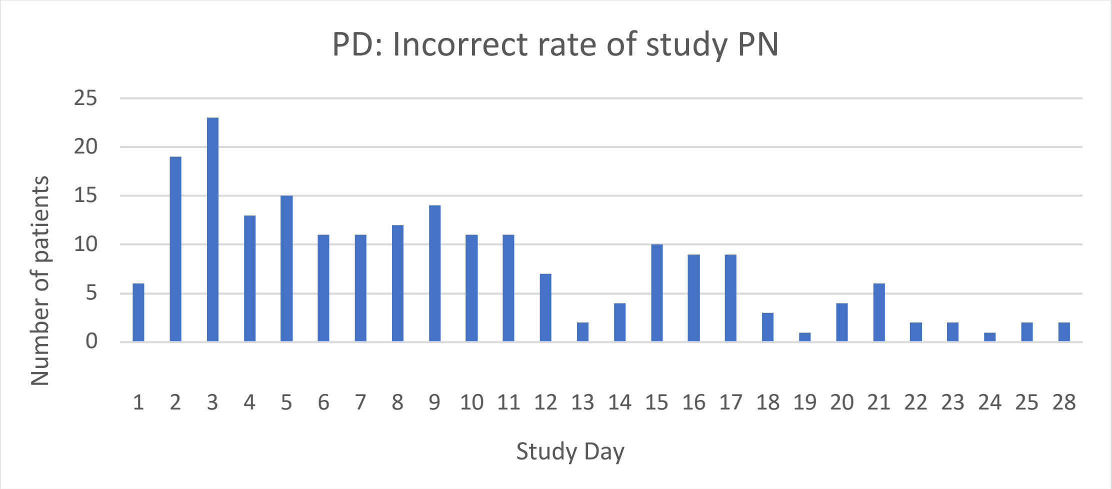

The consequences of these PD were as follows:

Consequence	N (%)
Study PN missed/withheld	145 (67%)
Other	39 (18%)
None	27 (13%)
Resulted in an AE	1 (0.5%)

ADDITIONAL FILE 10. DATA ON INTERVENTION CHARACTERISTICS AND DELIVERY

Across the study period, all intervention patients (n=119) and 17 (14%) patients in usual care received PN. There was no difference in the number of patients who received EN or resumed eating, nor the amount of energy or protein provided via these sources between groups (Additional Table 4). Usual hospital nutrition supplements were provided to 49 (41%) patients in intervention and 72 (61%) patients in usual care, providing similar energy and protein amounts (Additional Table 2). Study supplements were provided to 78 (66%) intervention patients providing 438 (204) kcal, 26 (12) g protein daily (20% (9%) of energy requirement and 25 (12%) protein requirement). From ICU to hospital discharge, more dietitian reviews were conducted in the intervention group (median 5 [3-7] vs 4 [2-6] in usual care), and time spent on the intervention was 0.7 [0.5-0.9] hours per occasion or 3 [2-5] hours in total.

ADDITIONAL TABLES

ADDITIONALTABLE 1. EXTENDED BASELINE PARTICIPANT CHARACTERISTICS

Variable	Tailored nutrition (n=119)	Usual care (n=118)
Height, cm, mean (SD)	173 (8)	173 (10)
Weight (actual), kg, mean (SD)	95 (25)	91 (24)
BMI, kg/m^2, n (%)		
Underweight (BMI <18.5 kg/m^2)	0 (0)	3 (3)
Normal weight (BMI 18.5-24.9 kg/m^2)	21 (18)	26 (22)
Overweight (BMI 25-29.9 kg/m^2)	42 (35)	38 (32)
Obese Class I (BMI 30.0-34.9 kg/m^2)	24 (20)	25 (21)
Obese Class II (BMI 35.0-39.9 kg/ m^2)	13 (11)	13 (11)
Obese Class III (BMI > 39.9 kg/m^2)	19 (16)	13 (11)
Location prior to ICU admission, n (%)		
Emergency Department	47 (40)	45 (38)
Operating theatre following elective surgery	23 (19)	22 (19)
Operating theatre following emergency surgery	23 (19)	15 (13)
Transfer from other ICU	9 (8)	17 (14)
Hospital Ward	12 (10)	10 (9)
Transfer from another hospital	5 (4)	9 (8)
Admitted to ICU following cardiac surgery, n (%)	24 (52)	25 (68)
Ethnicity recorded[b] (NZ participants only), n (%)	(n=33)	(n=32)
European	17 (52)	12 (38)
Māori	4 (12)	12 (38)
Middle Eastern/Latin American/African	0 (0)	0 (0)
Pacific peoples	9 (27)	7 (22)
Asian	3 (9)	1 (3)
Days from ICU admission to randomisation, days, median [IQR]	4 [3-5]	4 [3-4]

EN energy prior to randomisation, kcal/kg, mean (SD)	28 (24)	29 (25)
25% or 50% glucose prior to randomisation, n (%)	22 (18)	15 (13)
Volume, 25% glucose, ml, median [IQR]	18 [0-280]	19 [0-137]
Volume 50% glucose, ml, median [IQR]	8 [0-50]	0 [0-50]
Patients who received Propofol > 6 hours prior to randomisation, n (%)	106 (89)	104 (88)
Total volume, ml, mean (SD)	1169 (833)	1029 (684)
Energy requirements met from EN prior to randomisation, %, mean (SD)	27 (22)	29 (25)
0%-39.9%, n (%)	78 (70)	76 (70)
40%-79.9%, n (%)	32 (29)	26 (24)

Continuous data presented as mean (standard deviation) or median [IQR] in accordance with underlying distribution.

[b] Ethnicity is as follows, European includes European not further defined, New Zealand European, Other European; Māori includes New Zealand Māori; Middle Eastern/Latin American/African includes Middle Eastern, Latin American/Hispanic or African; Pacific peoples includes Pacific Island not further defined, Samoan, Cook Island Māori, Tongan, Niuean, Tokelauan, Fijian or Other Pacific Islands; Asian includes Asian not further defined, Southeast Asian, Chinese, Indian, Other Asian; Other includes Other ethnicity or unknown/didn't know.

Abbreviations: BMI body mass index; SD standard deviation; IQR interquartile range; EN enteral nutrition.

ADDITIONAL TABLE 2. ENERGY DELIVERY FROM NUTRITION AND NON-NUTRITION SOURCES OVER 28 DAYS AND BY LOCATION

Variable	Tailored nutrition (n=119)	Usual care (n=118)	Difference (95% CI)
**Daily energy provision from all sources (nutrition and non-nutrition energy)**			
Total energy delivery, kcal	1846±33	1527±33	318 (227 to 409)
Total energy, kcal/kg/CBW	22±0.3	19±0.3	3.21 (2.25 to 4.17)
Total energy, kcal/kg/actual	20±0.4	18±0.4	2.55 (1.46 to 3.63)
**ICU**			
Total energy delivery, kcal	1896±35	1635±37	261 (162 to 361)
Total energy, kcal/kg/CBW	23±0.4	21±0.4	2.37 (1.31 to 3.44)
Total energy, kcal/kg/actual	21±0.4	19±0.4	1.74 (0.55 to 2.92)
**Ward***			
Total energy delivery, kcal	1795±43	1420±43	375 (257 to 494)
Total energy, kcal/kg/CBW	22±0.5	18±0.5	4.05 (2.73 to 5.36)
Total energy, kcal/kg/actual	20±0.5	16±0.5	3.36 (1.99 to 4.73)

* Data on propofol and glucose is not included in the energy delivery as it was not collected on the ward.

ADDITIONAL TABLE 3. ENERGY AND PROTEIN PROVISION FROM EACH MODE OF NUTRITION OVER 28 DAYS

Variable	Tailored nutrition (n=119)	Usual care (n=118)
**Daily energy and protein provision from EN**		
Patients who received EN, n (%)	118 (99)	116 (98)
Days EN was received for, median [IQR]	12 [6-22]	10 [5-21]
Daily delivery of energy from EN, kcal, mean (SD)	1308 (460)	1332 (394)
Proportion of study energy requirement, %, mean (SD)	63 (21)	66 (18)
Daily delivery of protein, g, mean (SD)	63 (22)	65 (21)
Proportion of protein requirements, %, mean (SD)	61 (21)	65 (20)
**Daily energy and protein provision from PN**		
Patients who received PN, n (%)	119 (100)	17 (14)
Days PN was received for, median [IQR]	5 [2-11]	5 [4-14]
Delivery of energy, kcal, mean (SD)	783 (317)	1099 (360)
Proportion of study energy requirement, %, mean (SD)	37 (13)	51 (15)
Daily delivery of protein, g, mean (SD)	61 (24)	72 (24)
Proportion of protein requirements, %, mean (SD)	58 (22)	66 (22)
**Daily energy and protein provision from food**		
Patients who received food, n (%)	80 (67)	87 (74)
Days food provided, median [IQR]	6 [4-8]	5 [3-7]
Delivery of energy, kcal, mean (SD)	706 (399)	687 (335)
Proportion of study energy requirement, %, mean (SD)	33 (19)	33 (17)
Daily delivery of protein, g, mean (SD)	33 (22)	32 (17)
Proportion of protein requirements, %, mean (SD)	32 (21)	32 (18)
**Daily energy and protein provision from non- INTENT supplements**		
Patients who received non-study supplements, n (%)	49 (41)	72 (61)
Days provided, median [IQR]	3 [2-4]	3 [2-5]

Delivery of energy, kcal, mean (SD)	468 (238)	401 (189)
Proportion of study energy requirement provided, %, mean (SD)	21 (11)	19 (9)
Delivery of protein, g, mean (SD)	21 (11)	20 (9)
Proportion of protein requirements provided, %, mean (SD)	20 (10)	20 (10)
**Daily energy and protein provision from INTENT supplements**		
Patients who received INTENT supplements, n (%)	78 (65)	-
Days provided, median [IQR]	4 [2-6]	-
Delivery of energy, kcal, mean (SD)	438 (204)	-
Proportion of study energy requirement provided, %, mean (SD)	20 (9)	-
Delivery of protein, g, mean (SD)	26 (12)	-
Proportion of protein requirements provided, %, mean (SD)	25 (12)	-
**Daily energy and protein provision from food, INTENT supplements and non-INTENT supplements**		
Patients who received oral nutrition from any source, n (%)	84 (71)	89 (75)
Days provided, median [IQR]	6 [4-8]	5 [3-7]
Delivery of energy, kcal, mean (SD)	1021 (456)	868 (393)
Proportion of study energy requirement provided, %, mean (SD)	47 (22)	42 (19)
Delivery of protein, g, mean (SD)	51 (24)	40 (19)
Proportion of protein requirements provided, %, mean (SD)	49 (24)	41 (21)

Continuous data presented as mean (standard deviation) or median [IQR] in accordance with underlying distribution. Abbreviations: EN enteral nutrition; PN parenteral nutrition; CBW calculated body weight; SD standard deviation; IQR Interquartile range. INTENT supplements refers to supplements provided as part of the INTENT protocol (detailed at SI5 and SI Figure 1). Non-INTENT supplements refers to usual supplements available at the participating hospital.

ADDITIONAL TABLE 4. DAILY ENERGY AND PROTEIN DELIVERY IN ICU ACCORDING TO SOURCE

Variable	Tailored nutrition (n=119)	Usual care (n=118)
**Daily energy provision from non-nutrition energy sources**		
Patients who received non-nutrition energy sources, n (%)	111 (93)	111 (94)
Days non-nutrition energy sources provided, median [IQR]	5 [3-9]	6 [3-8]
Delivery of energy, kcal, mean (SD)	233 (155)	234 (138)
Proportion of energy, %, mean (SD)	11 (7)	12 (7)
**Daily energy and protein provision from EN**		
Patients who received EN, n (%)	118 (99)	116 (98)
Days EN was received for, median [IQR]	9 [5-18]	7 [4-17]
Daily delivery of energy from EN, kcal, mean (SD)	1295 (464)	1330 (393)
Proportion of study energy requirement, %, mean (SD)	63 (22)	66 (18)
Daily delivery of protein, g, mean (SD)	63 (23)	66 (21)
Proportion of protein requirements, %, mean (SD)	60 (22)	65 (20)
**Daily energy and protein provision from PN**		
Patients who received PN, n (%)	119 (100)	16 (14)
Days PN was received for, median [IQR]	5 [2-12]	4 [2-11]
Delivery of energy, kcal, mean (SD)	778 (314)	1057 (355)
Proportion of study energy requirement, %, mean (SD)	37 (13)	50 (16)
Daily delivery of protein, g, mean (SD)	61 (24)	71 (26)
Proportion of protein requirements, %, mean (SD)	58 (22)	65 (24)
**Daily energy and protein provision from food**		
Patients who received food, n (%)	61 (51)	56 (48)
Days food provided, median [IQR]	3 [2-5]	2 [1-4]
Delivery of energy, kcal, mean (SD)	510 (403)	389 (281)
Proportion of study energy requirement, %, mean (SD)	24 (19)	20 (16)
Daily delivery of protein, g, mean (SD)	24 (22)	17 (15)

Proportion of protein requirements, %, mean (SD)	22 (19)	18 (17)
**Daily energy and protein provision from non- INTENT supplements**		
Patients who received non-study supplements, n (%)	21 (18)	34 (29)
Days provided, median [IQR]	2 [1-3]	2 [1-3]
Delivery of energy, kcal, mean (SD)	370 (205)	372 (187)
Proportion of study energy requirement provided, %, mean (SD)	18 (9)	19 (10)
Delivery of protein, g, mean (SD)	17 (10)	19 (9)
Proportion of protein requirements provided, %, mean (SD)	16 (10)	19 (11)
**Daily energy and protein provision from INTENT supplements**		
Patients who received INTENT supplements, n (%)	51 (43)	-
Days provided, median [IQR]	2 [2-4]	-
Delivery of energy, kcal, mean (SD)	367 (210)	-
Proportion of study energy requirement provided, %, mean (SD)	17 (10)	-
Delivery of protein, g, mean (SD)	22 (13)	-
Proportion of protein requirements provided, %, mean (SD)	20 (11)	-
**Daily energy and protein provision from food, INTENT supplements and non-INTENT supplements**		
Patients who received oral nutrition from any source, n (%)	66 (55)	57 (48)
Days provided, median [IQR]	3 [2-5]	2 [1-4]
Delivery of energy, kcal, mean (SD)	695 (476)	535 (362)
Proportion of study energy requirement provided, %, mean (SD)	33 (24)	27 (20)
Delivery of protein, g, mean (SD)	35 (25)	25 (19)
Proportion of protein requirements provided, %, mean (SD)	32 (23)	25 (21)

Continuous data presented as mean (standard deviation) or median [IQR] in accordance with underlying distribution. Abbreviations: CBW calculated body weight; EN enteral nutrition; PN parenteral nutrition; SD standard deviation; IQR Interquartile range. INTENT supplements refers to supplements provided as part of the INTENT protocol (detailed at SI5 and SI Figure 1). Non-INTENT supplements refers to usual supplements available at the participating hospital.

ADDITIONAL TABLE 5. DAILY ENERGY AND PROTEIN DELIVERY ON THE WARD ACCORDING TO SOURCE*

Variable	Tailored nutrition (n=119)	Usual care (n=118)
**Daily energy and protein provision from EN**		
Patients who received EN, n (%)	51 (58)	48 (52)
Days EN was received for, median [IQR]	5 [3-11]	5 [3-10]
Daily delivery of energy from EN, kcal, mean (SD)	1479 (579)	1400 (639)
Proportion of study energy requirement, %, mean (SD)	68 (25)	64 (28)
Daily delivery of protein, g, mean (SD)	69 (26)	65 (30)
Proportion of protein requirements, %, mean (SD)	67 (26)	65 (31)
**Daily energy and protein provision from PN**		
Patients who received PN, n (%)	8 (9)	2 (2)
Days PN was received for, median [IQR]	1.5 [1-3]	2.5 [2-3]
Delivery of energy, kcal, mean (SD)	1059 (700)	1854 (1180)
Proportion of study energy requirement, %, mean (SD)	44 (27)	71 (33)
Daily delivery of protein, g, mean (SD)	65 (34)	83 (21)
Proportion of protein requirements, %, mean (SD)	56 (30)	72 (10)
**Daily energy and protein provision from food**		
Patients who received food, n (%)	70 (79)	79 (85)
Days food provided, median [IQR]	4 [2-5]	3 [2-5]
Delivery of energy, kcal, mean (SD)	830 (416)	838 (367)
Proportion of study energy requirement, %, mean (SD)	39 (21)	40 (20)
Daily delivery of protein, g, mean (SD)	39 (21)	39 (19)
Proportion of protein requirements, %, mean (SD)	38 (22)	40 (31)
**Daily energy and protein provision from non- INTENT supplements**		
Patients who received non-study supplements, n (%)	44 (50)	63 (68)
Days provided, median [IQR]	2 [1-3]	2 [2-4]
Delivery of energy, kcal, mean (SD)	510 (274)	417 (206)

Proportion of study energy requirement provided, %, mean (SD)	23 (13)	19 (10)
Delivery of protein, g, mean (SD)	22 (12)	20 (10)
Proportion of protein requirements provided, %, mean (SD)	21 (11)	20 (11)
**Daily energy and protein provision from INTENT supplements**		
Patients who received INTENT supplements, n (%)	64 (73)	-
Days provided, median [IQR]	2 [2-4]	-
Delivery of energy, kcal, mean (SD)	483 (217)	-
Proportion of study energy requirement provided, %, mean (SD)	22 (9)	-
Delivery of protein, g, mean (SD)	29 (13)	-
Proportion of protein requirements provided, %, mean (SD)	28 (13)	-
**Daily energy and protein provision from food, INTENT supplements and non-INTENT supplements**		
Patients who received oral nutrition from any source, n (%)	74 (84)	81 (87)
Days provided, median [IQR]	4 [2-5]	3 [2-5]
Delivery of energy, kcal, mean (SD)	1297 (549)	1044 (438)
Proportion of study energy requirement provided, %, mean (SD)	60 (25)	49 (23)
Delivery of protein, g, mean (SD)	64 (29)	48 (21)
Proportion of protein requirements provided, %, mean (SD)	63 (28)	49 (23)

Continuous data presented as mean (standard deviation) or median [IQR] in accordance with underlying distribution. Abbreviations: CBW calculated body weight; EN enteral nutrition; PN parenteral nutrition; SD standard deviation; IQR Interquartile range. INTENT supplements refers to supplements provided as part of the INTENT protocol (detailed at SI5 and SI Figure 1). Non-INTENT supplements refers to usual supplements available at the participating hospital.

* Data only collected 3 times per week on the ward.

ADDITIONAL TABLE 6. DAILY CLINICAL INFORMATION IN ICU

Variable	Tailored nutrition (n=119)	Usual care (n=118)
Patient received 25% glucose during stay, n (%)	8 (7)	4 (3)
Number of days patient received 25% Glucose, median [IQR]	1 [1-2]	1 [1-2]
Volume of 25% Glucose, ml mean (SD)	50 (44)	101 (14)
Patient received 50% glucose during stay, n (%)	1 (1)	7 (6)
Number of days patient received 50% Glucose, median [IQR]	1 [1-1]	1 [1-2]
Volume of 50% Glucose, ml, mean (SD)	15 (.)	80 (53)
Patient received Propofol during stay, n (%)	111 (93)	110 (93)
Number of days patient received Propofol, median [IQR]	5 [3-8]	5 [3-8]
Volume of Propofol, ml, mean (SD)	212 (141)	212 (127)
Patient received Insulin during stay, n (%)	62 (52)	61 (52)
Number of days patient received Insulin, median [IQR]	8 [4-18]	5 [3-10]
Units of Insulin, mean (SD)	60 (40)	58 (56)
Patient received Prokinetics during stay, n (%)	74 (62)	68 (58)
Number of days patient received Prokinetics, median [IQR]	4 [2-8]	4 [2-6]
Patient had Fasting/Interruption during stay, n (%)	112 (94)	113 (96)
Number of days patient had Fasting/Interruption, median [IQR]	5 [3-8]	4 [2-7]
Patient received RRT during ICU, n (%)	37 (31)	39 (33)
Days of Renal Replacement, median [IQR]	7 [5-16]	7 [3-16]
Number of patients with largest gastric volume recorded, n (%)	106 (89)	108 (92)
Number of days largest gastric volume recorded, median [IQR]	7 [4-12]	6 [4-11]
Largest gastric volume recorded, ml, mean (SD)	114 (104)	109 (97)
Morning Blood glucose was recorded, n (%)	119 (100)	118 (100)
Days morning blood glucose was recorded, median [IQR]	11 [6-19]	9 [6-18]
Morning blood glucose, mmol, mean (SD)	8.8 (1.8)	8.6 (1.7)
Blood glucose was below 2.1, n (%)	0 (0)	1 (1)
Number of times blood glucose was below 2.1, median [IQR]	-	3 [3-3]

Continuous data presented as mean (standard deviation) or median [IQR] in accordance with underlying distribution. Abbreviations: RRT Continuous Renal Replacement Therapy; SD standard deviation; IQR interquartile range.

ADDITIONAL TABLE 7. OUTCOME DATA

Variable	Tailored nutrition (n=119)	Usual care (n=118)
**Duration of Invasive Mechanical Ventilation to Day 28 (days), median [IQR]**		
All patients	9 [6-15]	8 [6-14]
Survivors	9 [6-14]	8 [6-13]
Non-survivors	12 [8-20]	13 [8-22]
**Lengths of stay (days), median [IQR]**		
ICU		
All patients	10 [6-17]	8 [5-16]
Survivors	10 [6-17]	8 [5-16]
Non-survivors	10 [5-20]	17 [9-18]
Hospital		
All patients	19 [14-35]	19 [13-32]
Survivors	22 [14-38]	20 [13-33]
Non-survivors	15 [6-21]	17 [10-25]
CLABSI n (%)	1 (1%)	1 (1%)

Abbreviations: IQR interquartile range; CLABSI central line-associated bloodstream infection.

ADDITIONAL FIGURES

ADDITIONAL FIGURE 1. FULL STUDY PROCESS

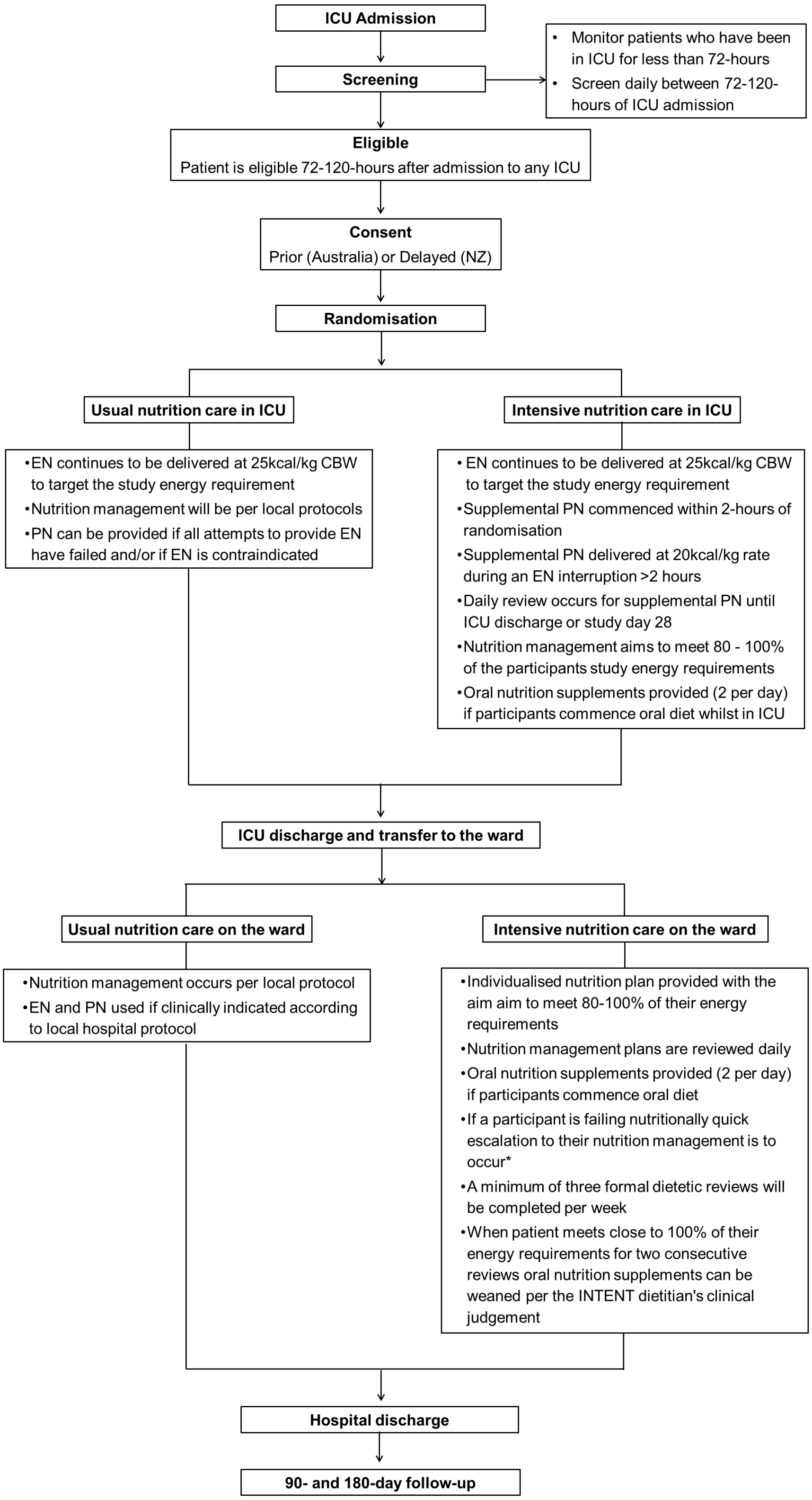

* Escalations to nutrition care were completed where a participant is failing to meet 80% of their study energy requirements. Such escalations may include, but are not limited to, prescribing an additional oral nutrition supplement(s), food fortification or modification of the diet prescription, and/or recommencement of EN or PN.

ADDITIONAL FIGURE 2. MANAGEMENT OF INTERVENTIONAL PN IN THE TAILORED NUTRITION GROUP

2A. Determining the rate of interventional PN delivery on the day of randomisation

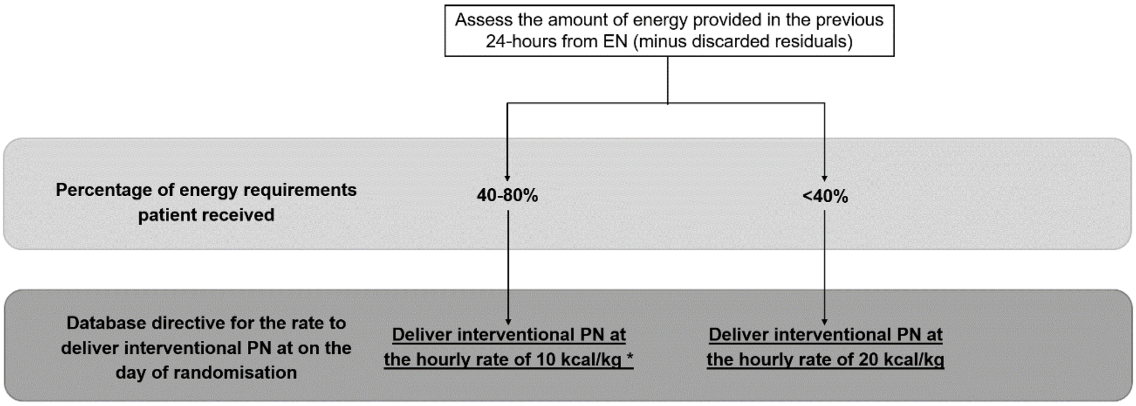

2B. Daily adjustment of interventional PN rate

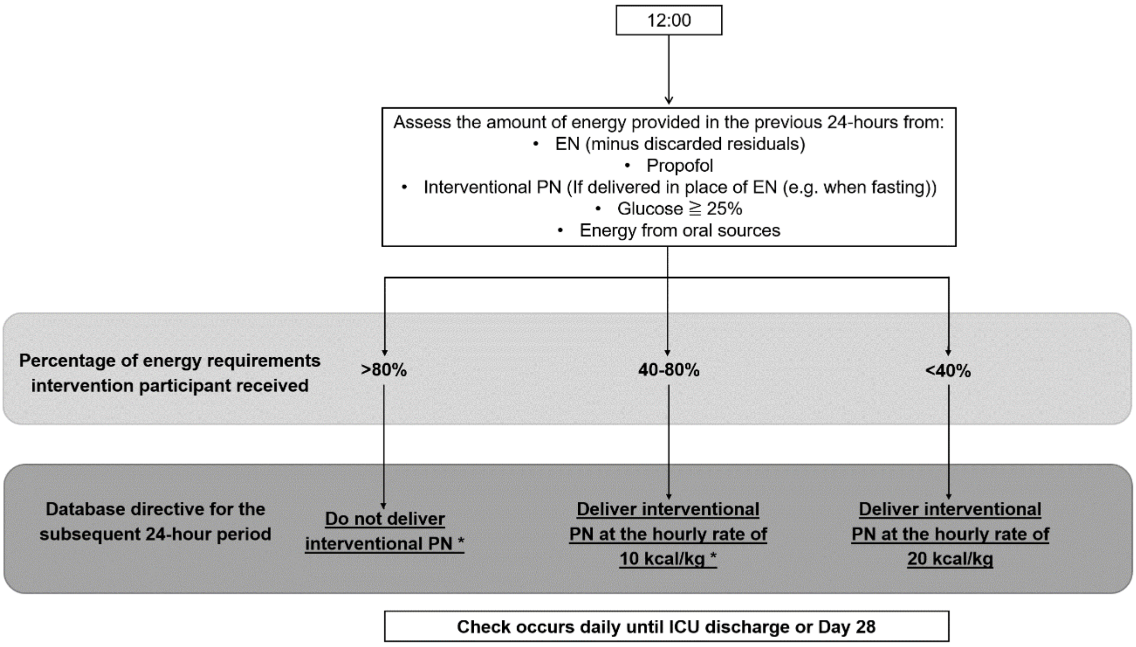

- In the event of an anticipated or actual interruption to EN for a period of 2-hours or more, interventional PN will be run at the hourly rate corresponding to 20 kcal/kg. If the patient is already receiving the highest rate of the intervention, there will be no change to the rate during the interruption. As soon as is practical, EN should be recommenced as per local protocol and the interventional PN returned to the rate determined per the midday assessment.

ADDITIONAL FIGURE 3. DELIVERY OF NUTRITION OVER 28 DAY STUDY PERIOD

3A. Daily energy delivery from nutrition (g/kg/CBW/day)

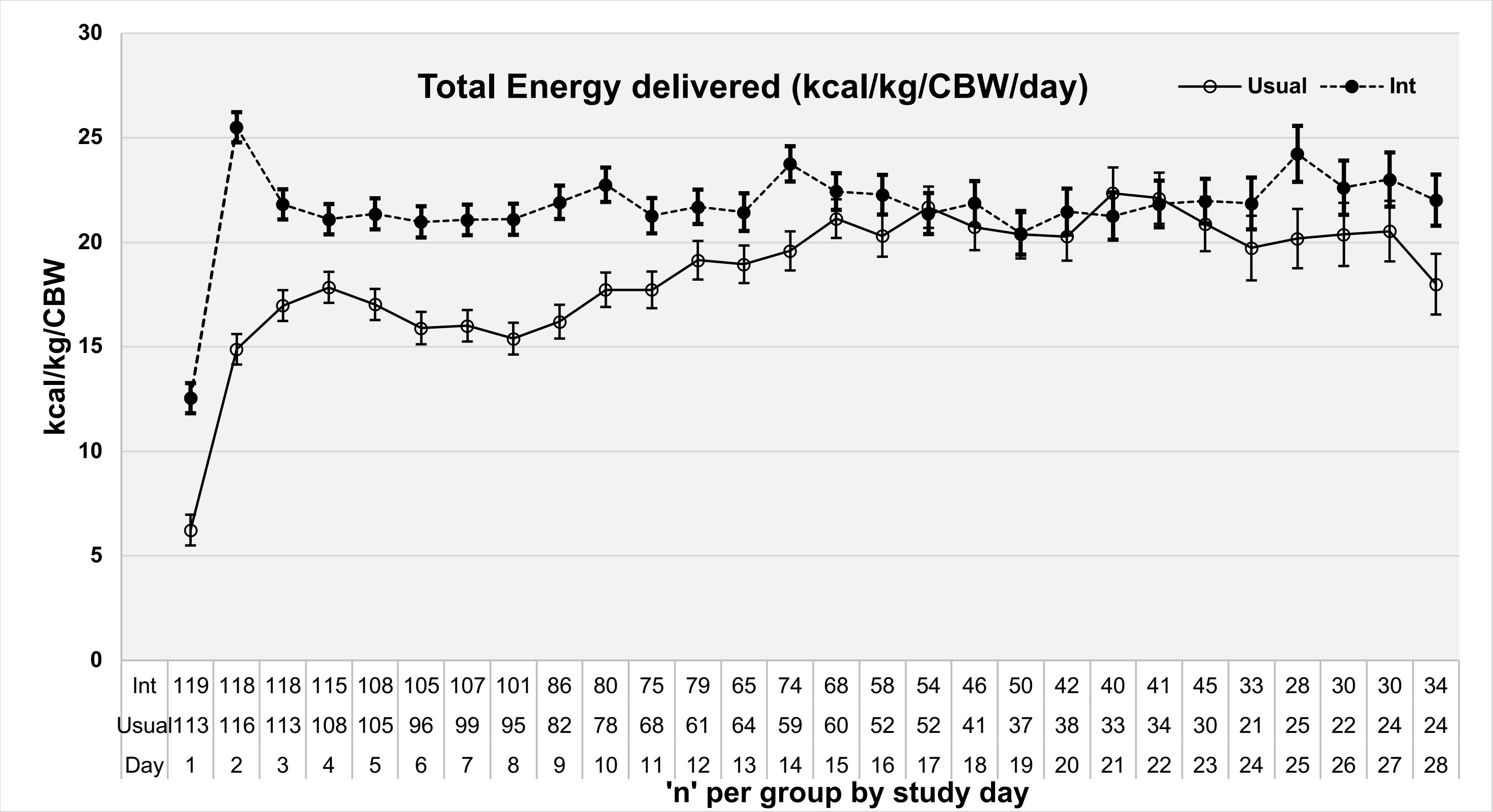

Int; Tailored nutrition; Usual: Usual care
Circles represent least square means with error bars representing 95% confidence interval
The overall difference between groups across all time points was p<0.0001.

3B. Daily protein delivery from nutrition (g/kg/CBW/day)

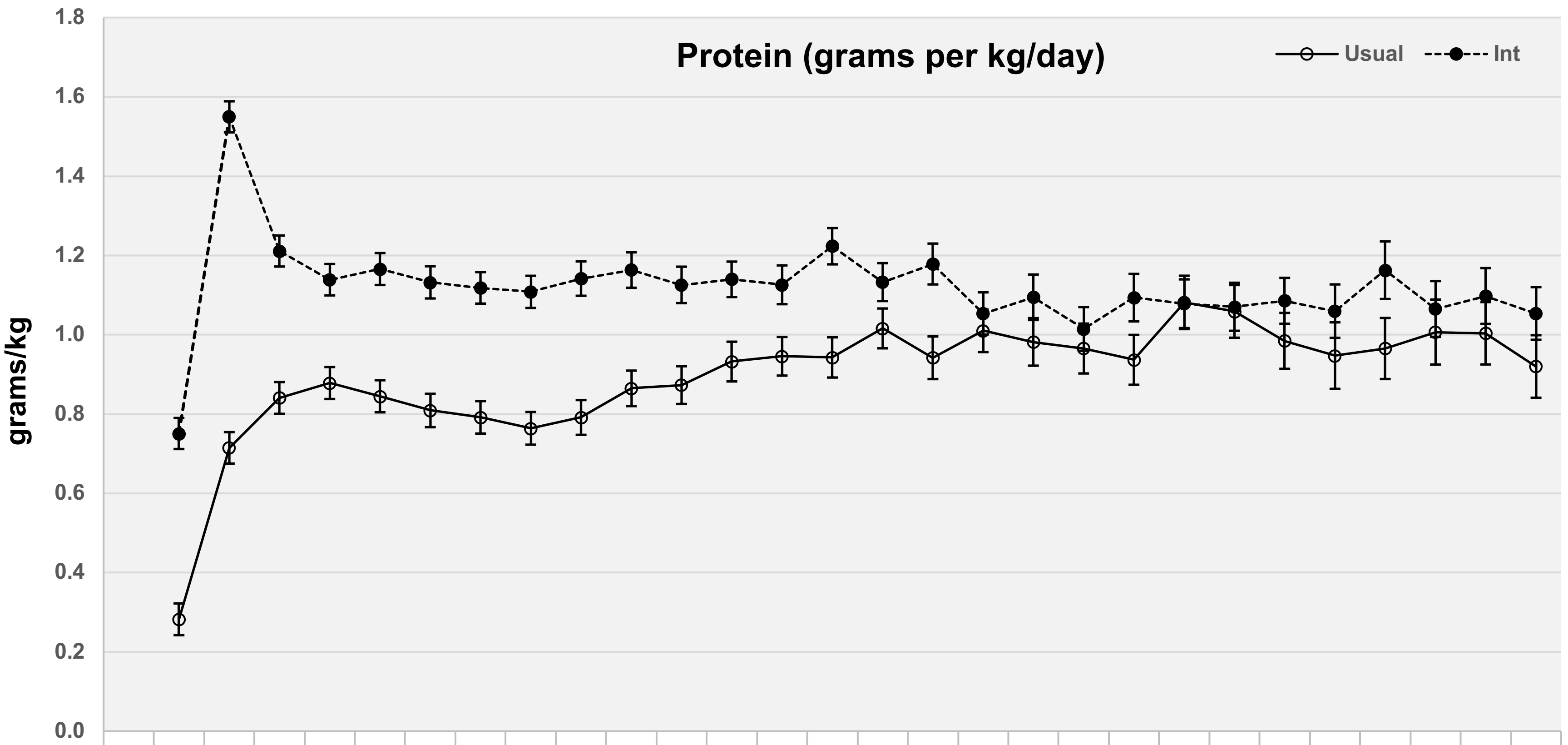

Int	119	118	118	115	108	105	107	101	86	80	75	79	65	74	68	58	54	46	50	42	40	41	45	33	28	30	30	34
Usual	114	117	114	109	106	97	100	96	83	79	69	62	65	60	61	53	53	42	38	38	33	34	30	21	25	22	24	24
Day	1	2	3	4	5	6	7	8	9	10	11	12	13	14	15	16	17	18	19	20	21	22	23	24	25	26	27	28

'n' per group by study day

Int; Tailored nutrition; Usual: Usual care
Circles represent least square means with error bars representing 95% confidence interval
The overall difference between groups across all time points was p<0.0001.

ADDITIONALFIGURE 4. MODE OF NUTRITION DELIVERY IN ICU BY DAY

4A. Tailored nutrition

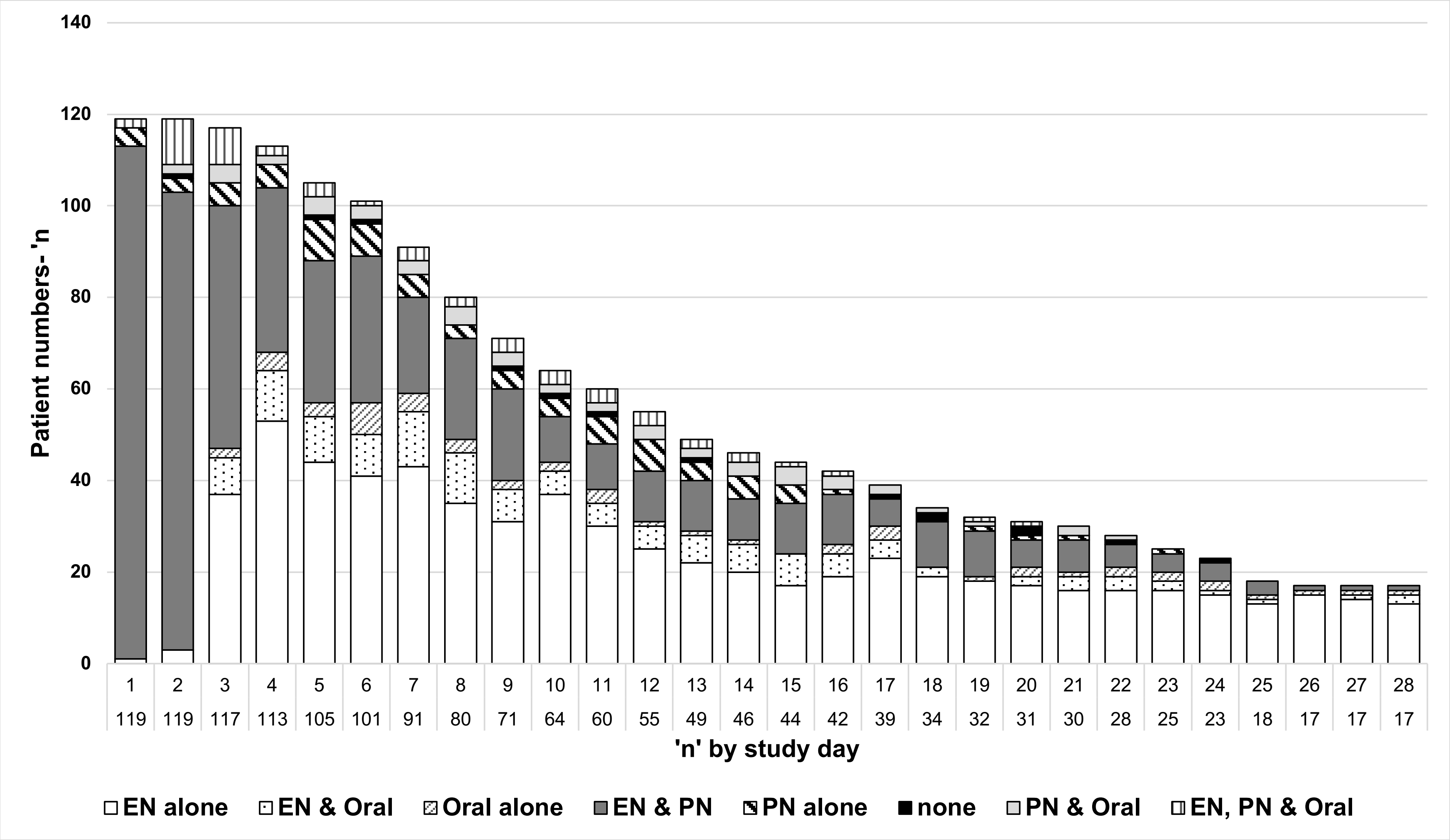

4B. ICU- Usual care

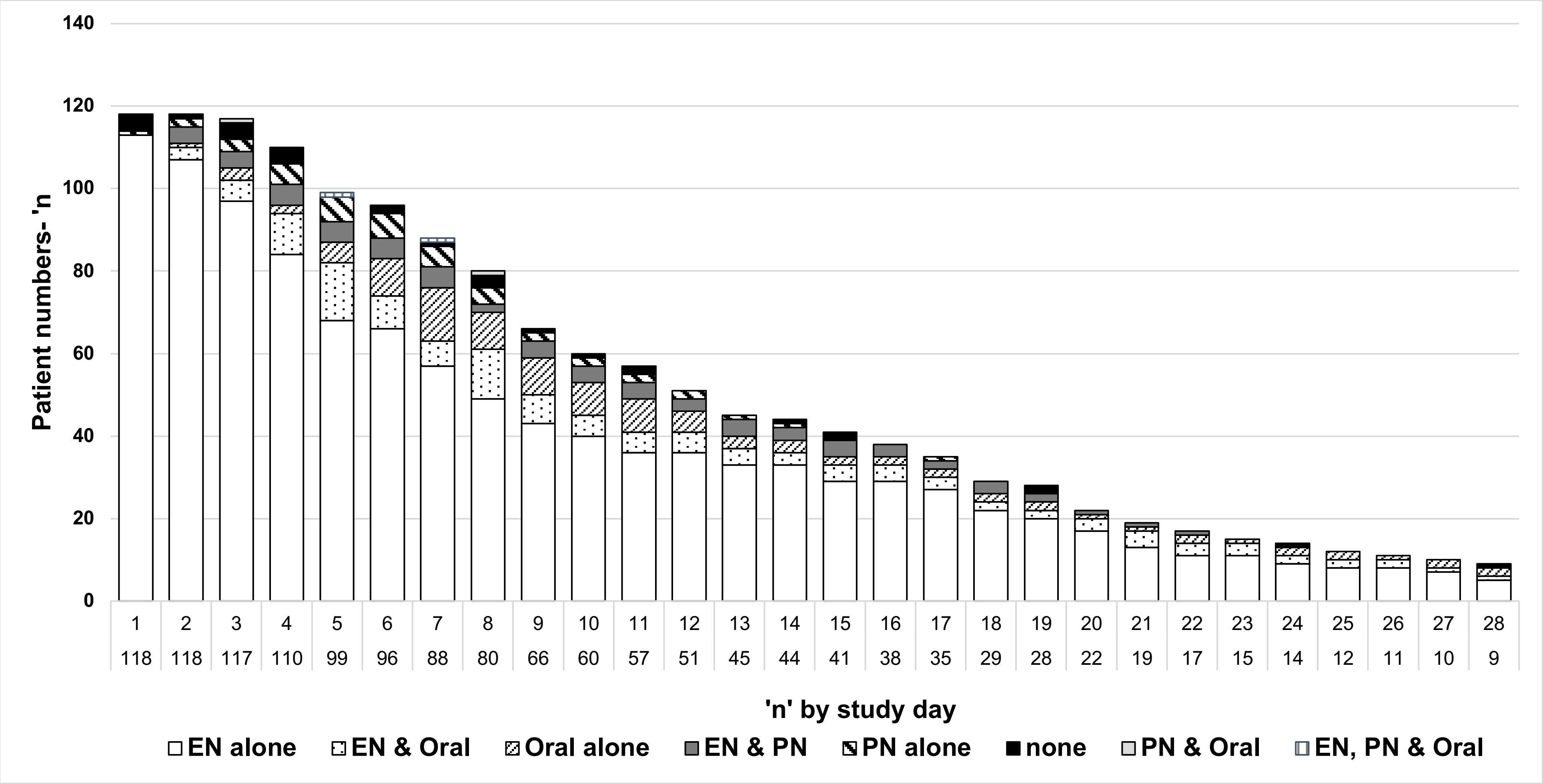

ADDITIONAL FIGURE 5. MODE OF NUTRITION DELIVERY ON THE WARD

5A. Tailored nutrition

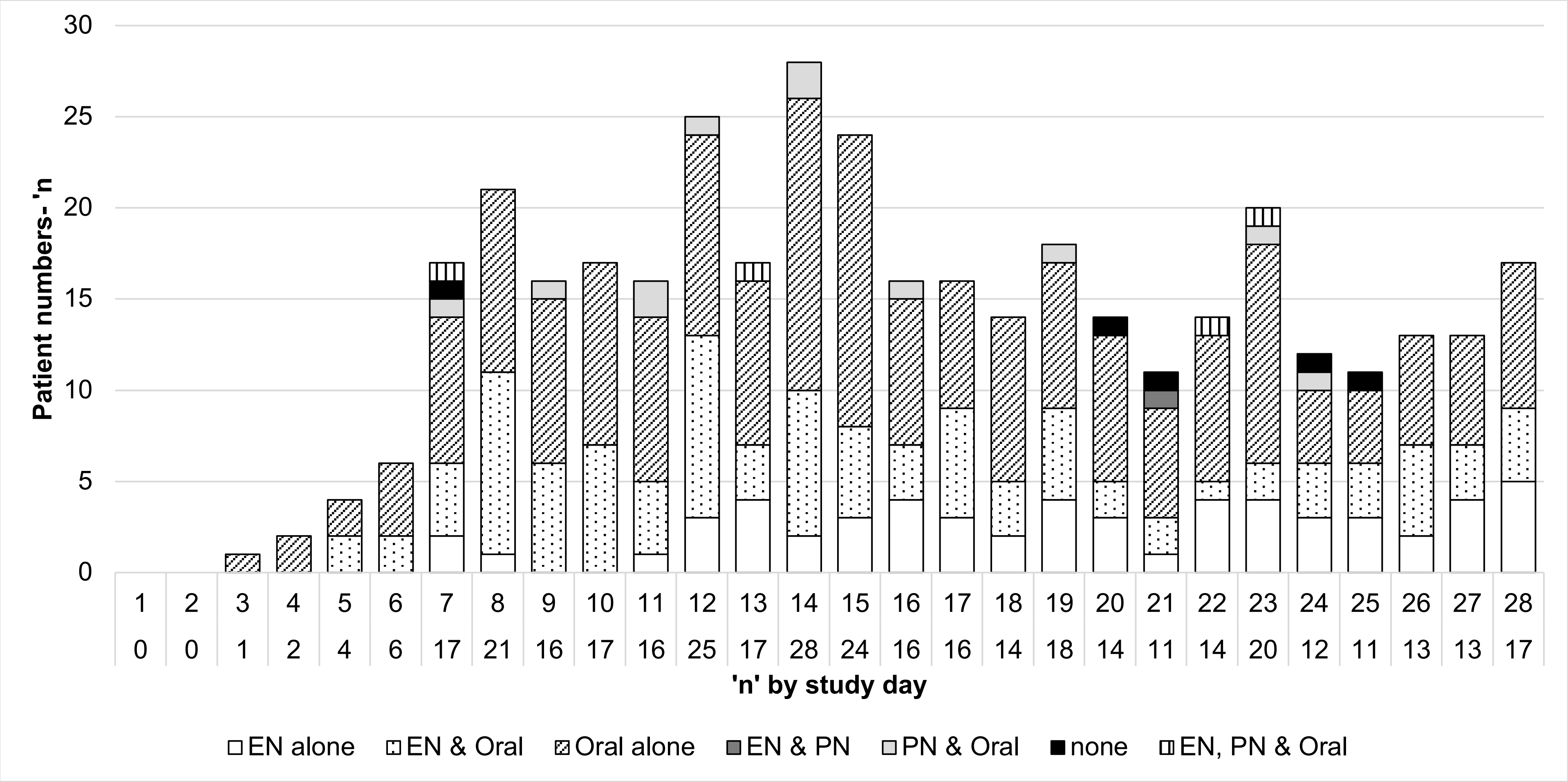

5B. Usual care

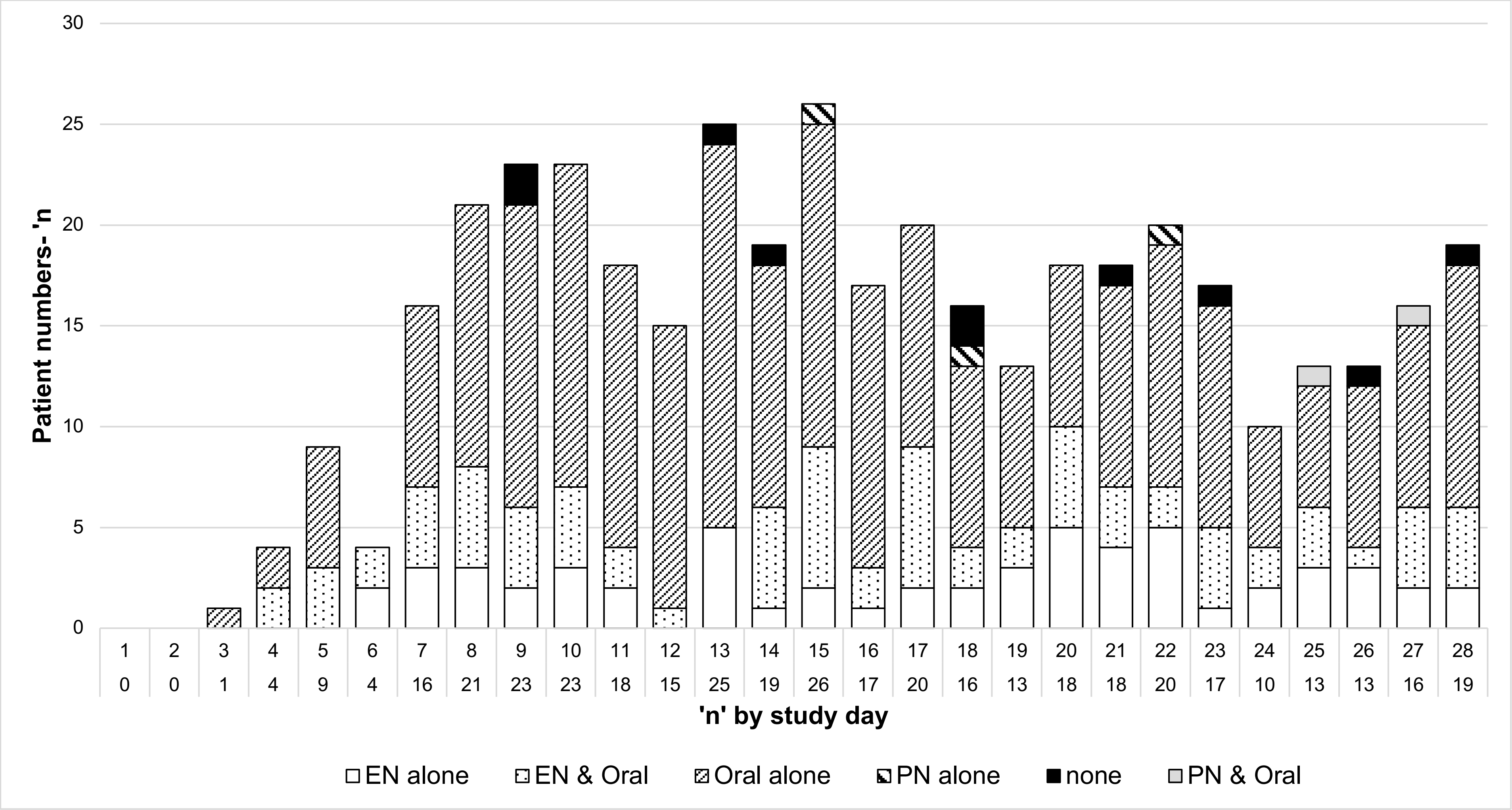

ADDITIONAL FIGURE 6. CUMULATIVE INCIDENCE PLOTS

For the following graphs: Intensive; Tailored nutrition; Usual: Usual care

6A. Days from randomisation to hospital discharge

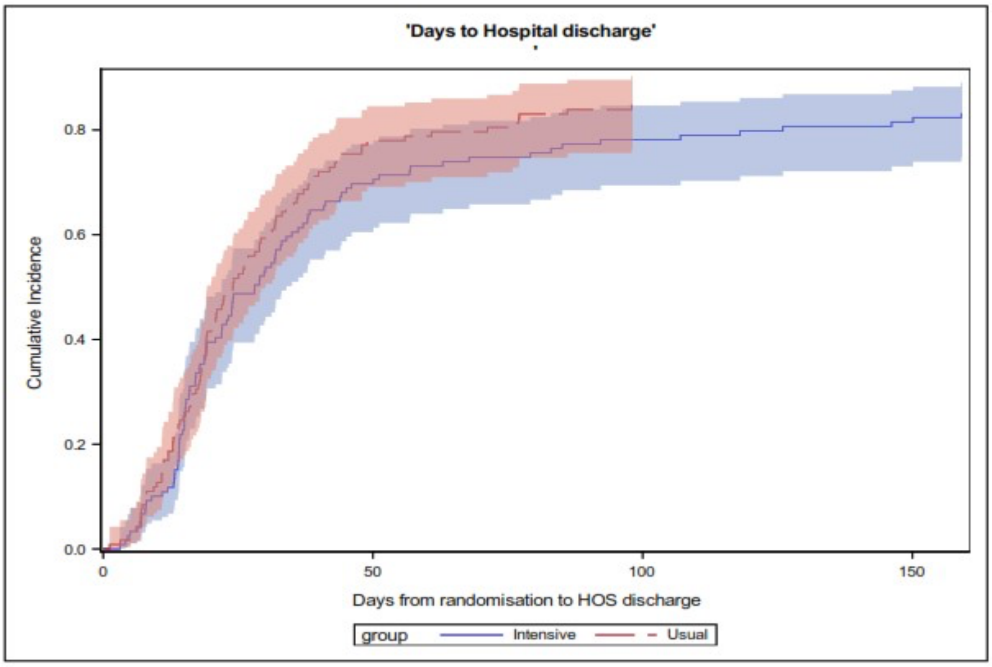

Hazard ratio (95%CI) (Intensive vs Usual); 0.89 (0.67-1.17).

6B. Days from randomisation to extubation from mechanical ventilation

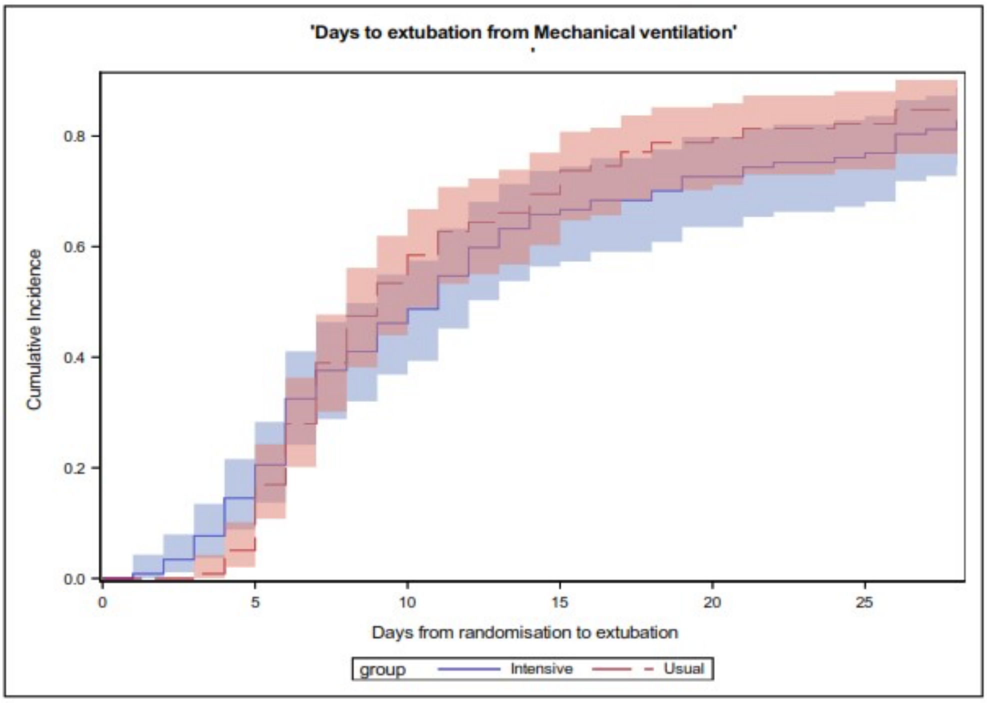

Hazard ratio (95%CI) (Intensive vs Usual); 0.92 (0.70-1.20).

6C. Days from randomisation to ICU discharge

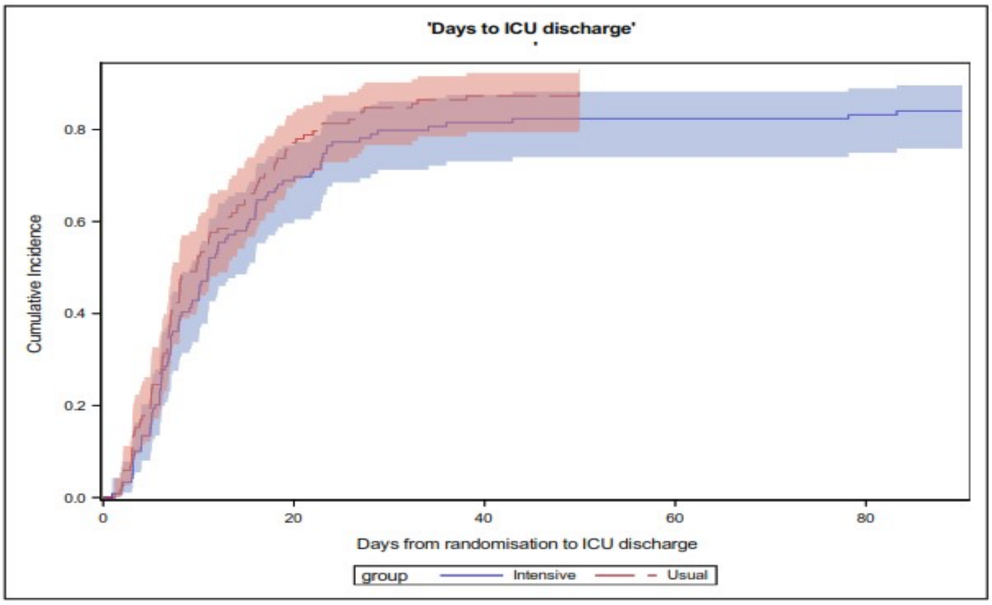

Hazard ratio (95%CI) (Intensive vs Usual); 0.84 (0.64-1.11).

6D. Days from randomisation until first blood stream infection

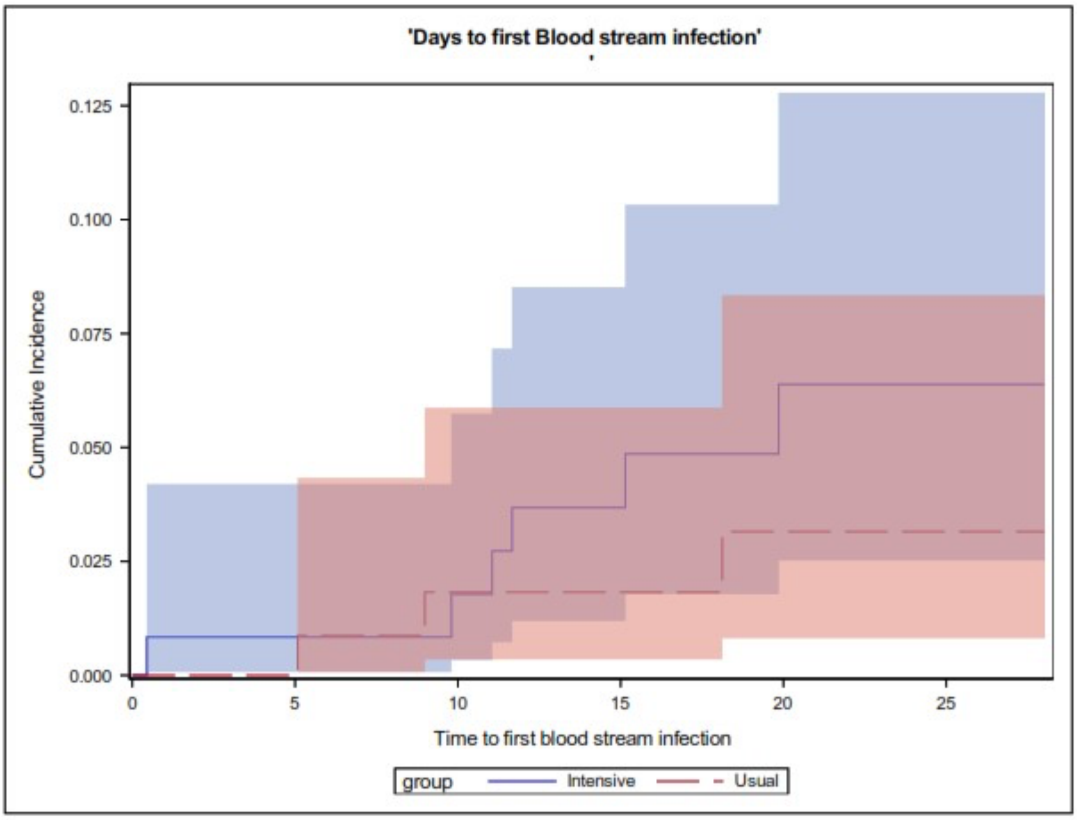

Hazard ratio (95%CI) (Intensive vs Usual); 1.99 (0.5-7.82).

ADDITIONAL FIGURE 7. PATIENT SURVIVAL IN DAYS FROM RANDOMISATION (CENSORED AT 250 DAYS)

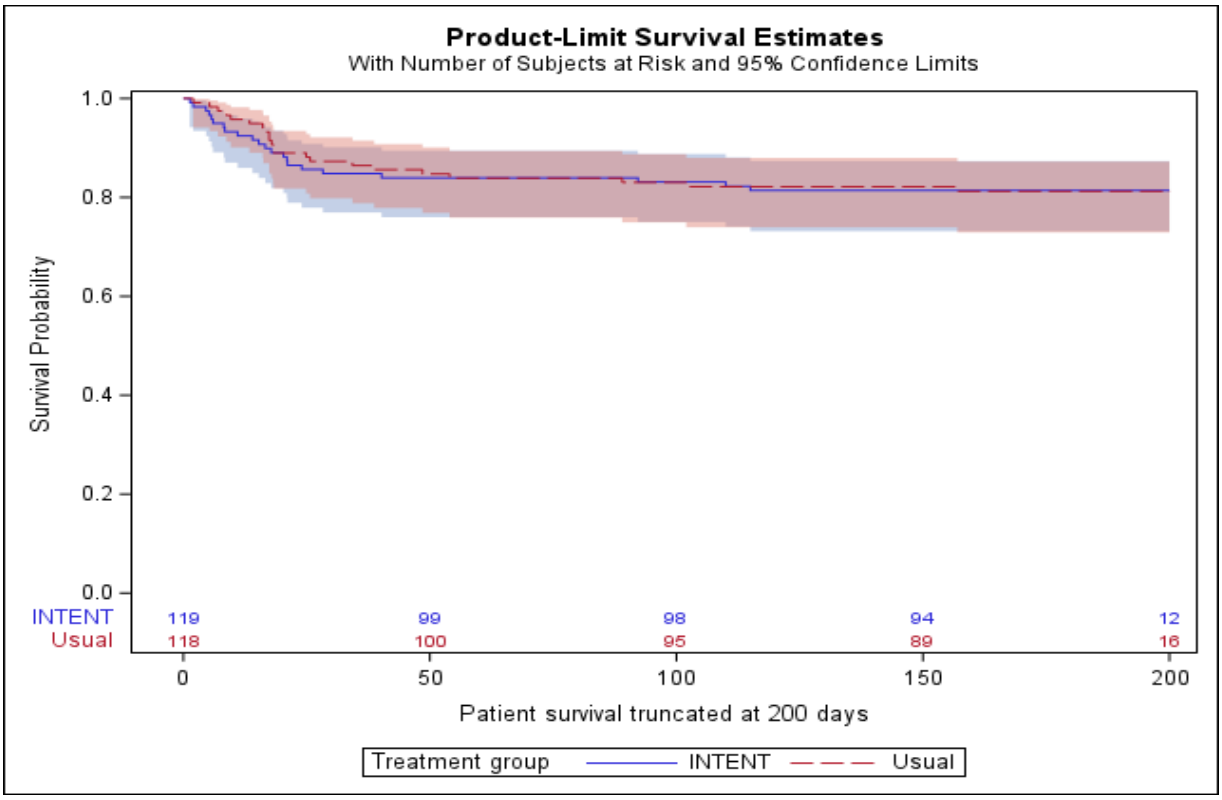

Raw Hazard Ratio for Intensive vs Usual HR 1.01 (0.56 – 1.83) p=0.96.

ADDITIONAL FIGURE 8. CLINICAL FRAILTY SCORE

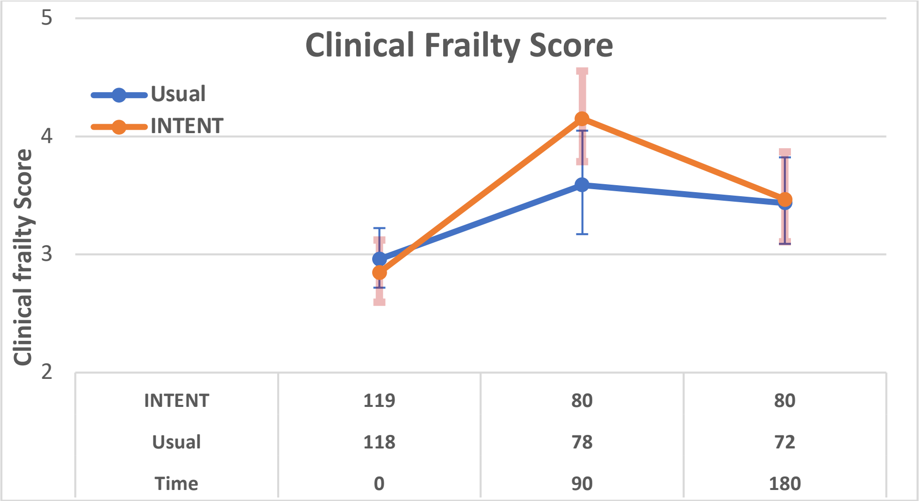

Frailty score was measured at baseline, day 90 and day 180.

Overall difference between groups (p=0.48).

ADDITIONAL FIGURE 9. PATIENT WEIGHT

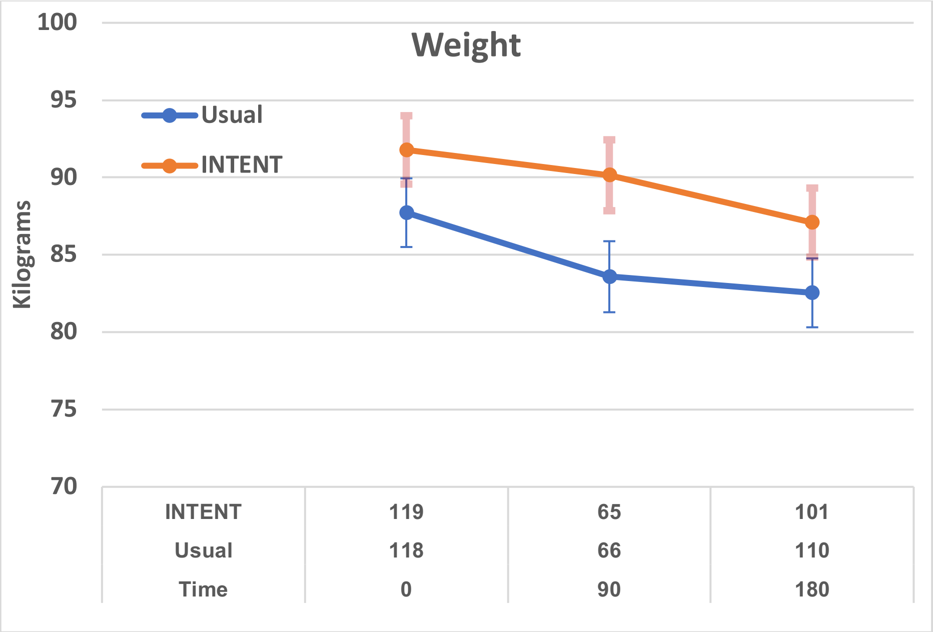

Weight was measured at baseline, day 90 and day 180.

Overall difference between groups (p=0.10).

ADDITIONAL FIGURE 10. PATIENT STATUS ACROSS THE 28 DAY STUDY PERIOD

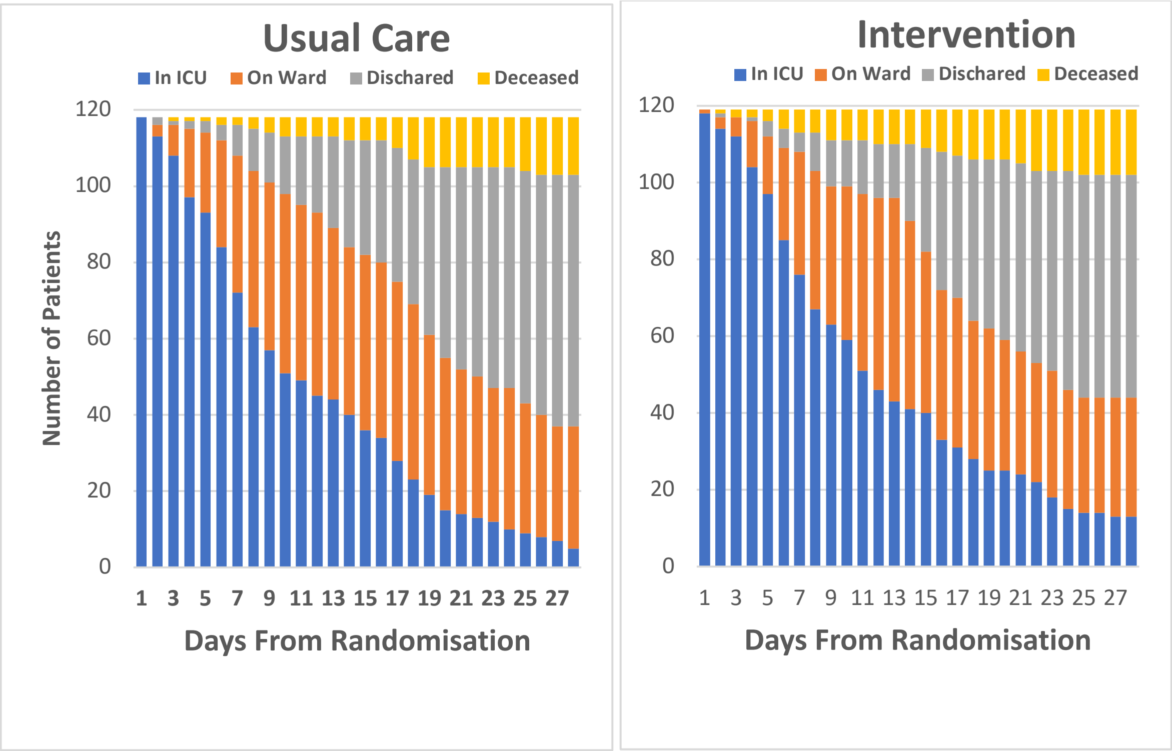

ADDITIONAL FIGURE 11. DAILY ENERGY DIFFERENCE (KCAL) FOR SPECIFIED SUB-GROUPS

Subgroup	Intensive nutrition	Usual nutrition	Mean difference [95% CI]	P interaction
	Number of patients			
All patients	119	118	313 [223-403]	
Sex				0.1
Male	83	78	412 [263 -561]	
Female	36	40	261 [158 -365]	
Age				0.55
≤ 60 yr	64	64	335 [220 -450]	
> 60 yr	55	54	284 [157 -410]	
APACHE 2 Score				0.49
≤ 16	69	53	331 [211-452]	
> 16	50	65	272 [149-396]	
Body mass index (kg/m2)				0.23
≤ 30	63	67	258 [145-372]	
> 30	56	51	358 [233-482]	
Clinical frailty score				0.14
≤ 3	77	68	263 [154-372]	
> 3	42	50	392 [254-530]	
Renal replacement at baseline				0.69
No	90	84	325 [223-428]	
Yes	29	34	287 [128-446]	
Cardiac surgery				0.25
No	95	93	338 [240-436]	
Yes	24	25	218 [34-402]	

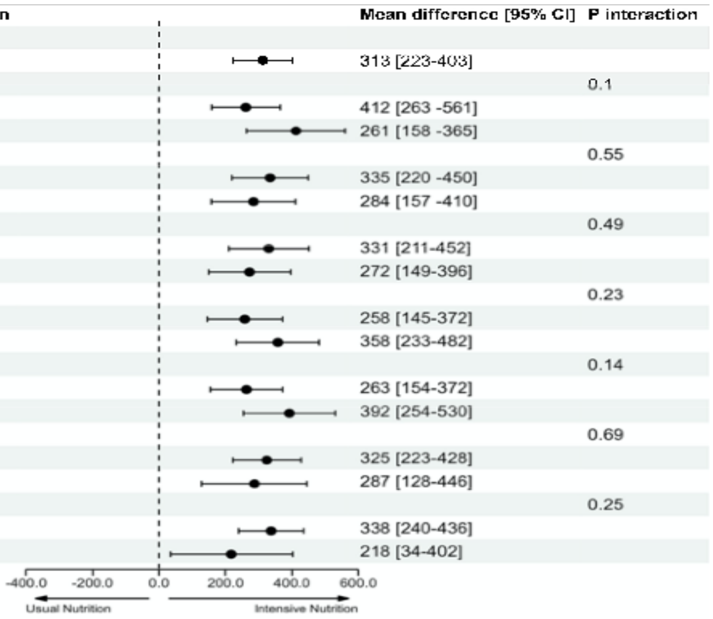

REFERENCES

1. Ridley EJ, Bailey M, Chapman M, Chapple LS, Deane AM, Hodgson C, et al., (2022) Protocol summary and statistical analysis plan for Intensive Nutrition Therapy comparEd to usual care iN criTically ill adults (INTENT): a phase II randomised controlled trial. BMJ Open 12: e050153
2. Care ACoSaQiH, (2015) Implementation Guide for Surveillance of Central Line Associated Bloodstream Infection DOI
3. Commission HQS, (2020) How to prevent central line associated bacteraemia (CLAB) DOI
4. Cook D, Lauzier F Fau - Rocha MG, Rocha Mg Fau - Sayles MJ, Sayles Mj Fau - Finfer S, Finfer S, Serious adverse events in academic critical care research
